# BCAA catabolism targeted therapy for heart failure with preserved ejection fraction

**DOI:** 10.7150/thno.105894

**Published:** 2025-05-24

**Authors:** Meng Wang, Zhao Liu, Shuxun Ren, Jinyun Zhu, Norihiko Morisawa, Geok Lin Chua, Xuewen Zhang, Yun Ka Wong, Liping Su, Ming Xiang Wong, Jieping Yang, Marc Titze Jens, Zhaoping Li, Haipeng Sun, Yibin Wang, Christoph D. Raul, Sanjiv J. Shah, Chen Gao, Yunxia Liu

**Affiliations:** 1 Signature Research Programme in Cardiovascular and Metabolic Disorders, DukeNUS Medical School, Singapore.; 2 National Heart Center of Singapore, Singapore.; 3 Department of Medicine, University of California, Los Angeles, CA, USA.; 4 NHC Key Laboratory of Hormones and Development, Tianjin Key Laboratory of Metabolic Diseases, Chu Hsien-I Memorial Hospital & Tianjin Institute of Endocrinology, Tianjin Medical University, Tianjin 300134, China.; 5 Department of Genetics and Computational Medicine Program, University of North Carolina at Chapel Hill, NC, USA.; 6 Division of Cardiology, Department of Medicine, Northwestern University Feinberg School of Medicine, IL, USA.; 7 Department of Pharmacology and Systems Physiology, University of Cincinnati, OH, USA.

**Keywords:** heart failure, HFpEF, BCAA metabolism, BT2, insulin resistance

## Abstract

**Rationale:**

Heart failure with preserved ejection fraction (HFpEF) is a major unmet medical need with limited effective treatments. A significant contributing factor to HFpEF, a multifactorial disease, is underlying metabolic dysfunction. While much of the prior research has been on glucose and fatty acid metabolic defects in the pathogenesis of HFpEF, other metabolic activities remain under investigated.

**Methods:**

System-based metabolomics and targeted mass spectrometry were employed to analyze serum and tissue samples from a deep-phenotyped human HFpEF cohort. A preclinical mouse model of HFpEF was developed by combined administration of a high-fat diet (HFD) and the nitric oxide (NO) synthase inhibitor N[w]-nitro-l-arginine methyl ester (L-NAME). The branched-chain amino acid (BCAA) catabolic activities were enhanced by genetic inactivation of branched-chain ketoacid-dehydrogenase kinase (BCKDK) or treatment with BT2 (3,6-dichlorobenzo[b]thiophene-2-carboxylic acid), a highly selective inhibitor of BCKDK. Cardiac function, myocardial remodeling and insulin signaling in the left ventricle were assessed across all experimental cohorts.

**Results:**

The systems-based metabolomics analysis of the deep-phenotyped HFpEF and non-HFpEF patients revealed that abnormal circulating BCAA levels were significantly associated with adverse outcomes. In the rodent model of HFpEF, significant impairment of BCAA catabolic activities in the heart and abnormal circulating BCAA levels were also observed. In adult mice, inducible knockout of BCKDK, the rate-limiting negative regulator of BCAA catabolic flux, markedly augmented BCAA catabolic activities. Compared with the controls, BCKDK inactivation blunted diastolic dysfunction, cardiac hypertrophy and myocardial remodeling in response to chronic treatment with HFD/L-NAME. This functional amelioration was associated with improved insulin signaling in the myocardium and reduced S-nitrosylation of cardiac proteins, without any impact on systemic blood pressure. Finally, pharmacological inhibition of BCKDK in HFpEF mice significantly reversed the diastolic dysfunction and cardiac hypertrophy associated with HFpEF.

**Conclusions:**

Our study provides the first proof-of-concept evidence that global catabolic impairment of BCAAs is an important pathogenic contributor and metabolic signature of HFpEF and restoring BCAA catabolic flux could be an efficacious therapeutic strategy for HFpEF.

## Introduction

Heart failure with preserved ejection fraction (HFpEF) accounts for more than 50% of heart failure cases and is arguably the most significant unmet medical need amongst cardiovascular diseases because of its poor outcomes and lack of effective therapies 1, 2. HFpEF is a heterogeneous disease often associated with multiple comorbidities and risk factors, including obesity, diabetes, hypertension and aging. HFpEF pathology is characterized by heart failure symptoms with a normal range of ejection fraction but profound defects in diastolic function, cardiac hypertrophy and fibrotic remodeling. The pathogenesis of HFpEF remains elusive but clearly involves cardiac metabolic remodeling in combination with other mechanical or neural-hormonal stresses 3-5. However, standard treatment strategies for heart failure with reduced ejection fraction (HFrEF), such as β-blockers, ACEi and spironolactone, fail to improve the clinical outcome for HFpEF patients 6-9. In contrast, studies on the outcomes of patients with anti-diabetic SGLT-2 inhibitors and anti-obesity GLP-1 receptor agonists have demonstrated exciting and unexpected benefits for HFpEF, highlighting the potential of metabolism-targeted therapy for this disease 10-14. However, despite the overall positive outcomes observed from the entire study population, subgroup analyses within these trials have also revealed reduced benefits for significant portions of patients with HFpEF 10, 15. Therefore, better stratification and new targeted therapies for HFpEF are still urgently needed.

The branched-chain amino acids (BCAAs), including leucine (Leu), isoleucine (Ile) and valine (Val), are essential amino acids primarily obtained from protein-containing foods 16. Previous research has reported that the BCAA catabolic pathway is highly enriched among the differentially expressed genes detected in both mouse and human failing hearts 17-19. Transcriptomic analysis of a large animal model of HFpEF has also revealed significant changes in the BCAA metabolic pathway 20. More recently, a metabolomic analysis in myocardial tissues from HFpEF patients has further revealed impaired BCAA catabolic activity in the human HFpEF hearts 21. Surprisingly, despite these lines of evidence supporting the notion that impaired BCAA catabolism is a conserved pathological feature associated with HFpEF, whether BCAA catabolism can serve as both a biomarker and a therapeutic target for HFpEF has never been directly demonstrated in the current literature.

BCAA homeostasis is tightly regulated by dietary intake, and BCAA catabolism primarily occurs in the mitochondria. The BCAA catabolic pathway includes reversible transamination to branched chain α-ketoacids (BCKAs) followed by irreversible BCKA oxidation catalyzed by the rate-limiting enzyme complex of BCKA dehydrogenase (BCKDH) 16, 22, BCKAs include keto-isocaproate (KIC), keto-isovalerate (KIV) and keto-methylvalerate (KMV). BCKDH enzymatic activity is tightly regulated by the phosphorylation of its E1α subunit (BCKDHA). BCKD kinase (BCKDK) inhibits BCKDH activity, thereby reducing BCAA catabolic flux, whereas the protein phosphatase PPC2m activates BCKDH, leading to an increase in BCAA catabolism 23, 24. In this study, we performed an unbiased systems-based metabolomics analysis in a deep-phenotyped human cohort of HFpEF and non-HFpEF patients. The results showed that BCAA and glucose were in the same metabolic module with the highest correlation with adverse outcomes in HFpEF patients. We further demonstrated that systemic catabolic defects in BCAA correlated with disease progression in a mouse model of HFpEF. Importantly, targeted enhancement of BCAA catabolic flux ameliorated the characteristic pathological features of HFpEF, including diastolic dysfunction, cardiac hypertrophy and remodeling. Mechanistically, we demonstrated that BCAA catabolic activation did not affect systemic blood pressure but markedly improved insulin signaling while reducing myocardial S-nitrosylation levels. These findings provide the first proof-of-concept evidence that BCAA catabolic defects are functionally important for metabolic remodeling and restoration of the BCAA catabolic flux is a potent therapeutic strategy for treating HFpEF.

## Materials and Methods

### Human Metabolites Analysis

Untargeted metabolomic analysis was performed on serum samples collected from a Northwestern cohort of HFpEF and control subjects, and their clinic characteristics were described in Supplemental Table 17 as reported by Culler et al 25. Metabolite Enrichment: Metabolites that were significantly different at an FDR of 5% by a Kruskal-Wallis test between control and HFpEF individuals were uploaded to the Overrepresentation tool on the MetaboAnalyst 4.0 server (https://currentprotocols.onlinelibrary.wiley.com/doi/10.1002/cpbi.86) and compared to KEGG pathways to search for enriched pathways associated with changes between HFpEF and control individuals. A total of 707 metabolites with a coefficient of variation greater than 7.5% were examined using Weighted Gene Co-expression Network Analysis (WGCNA) (https://bmcbioinformatics.biomedcentral.com/articles/10.1186/1471-2105-9-559) using the standard pipeline as described. Briefly, correlations were calculated between each pair of genes using the biweight midcorrelation. Next, these correlations were soft-thresholded (values raised to a power of 3) to achieve a closer fit to a scale-free topology. The modified correlations were then converted to a measure of topological overlap as follows:



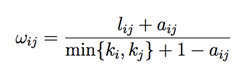



where w_ij is the topological overlap between genes i and j, l_ij is the sum across all other genes u of the product of the weighted correlations between genes i and u and j and u, a_ij is the weighted correlation between genes i and j, and k_i is the total sum of all weighted correlations between gene i and all other genes in the dataset. The resulting topological overlap matrix is then subjected to hierarchical clustering, and modules are determined using the dynamic tree cut algorithm. The first principal component of each module (termed the 'eigengene') is determined and correlated with phenotypic traits to identify modules that are strongly associated with specific phenotypes of interest.

### Animals and Tamoxifen Treatment

All mice were raised and kept in the animal facility with a 12-hour light/dark cycle at Duke-NUS Medical School, with an approved protocol of the SingHealth Institutional Animal Care and Use Committee, Singapore. The *bckdk*^flox/flox^-ubiqutin C Cre-ERT2 mice (BCKDK-uKO) were generated by crossing the previously established *bckdk*^flox/flox^ mice 26 with a transgenic line (B6. Cg-*Ndor1^Tg(UBC-cre/ERT2)1Ejb^*/1J, strain number 007001, from Jacksonville, FL, USA), where the human ubiquitin C promoter drives the expression of the tamoxifen-inducible Cre transgene. The genotype results were confirmed by genomic DNA PCR (Table S1). At two months of age, a tamoxifen diet was administered for two weeks, followed by one week of washout. Next, cardiac function was evaluated, and the global inactivation of the *Bckdk* gene was confirmed by Real-Time PCR and Western blotting at both mRNA and protein levels. The HFpEF mouse model was induced by administration of a 60% high-fat diet (Research Diets, D12492i) plus Nω-Nitro-L-arginine methyl ester (L-NAME: 0.5 mg/mL, Sigma Aldrich) in the drinking water as described previously 27.

### Echocardiogram

The mice were anesthetized and maintained under anesthesia with a mixture of 2% isoflurane. Cardiac function was measured using the high-frequency ultrasound system Vevo F2 (FUJIFILM VisualSonics), which is equipped with a 57-MHz linear transducer (UHF57x) for precise imaging. The left ventricular ejection fraction (LVEF) was measured using the Teichholz formula, and images were acquired in short-axis view (SAX) in M-mode. The E/A ratio, E/e' ratio and IVRT (isovolumetric relaxation time) were obtained via an apical 4-chamber view with pulse wave Doppler (PWD) and tissue Doppler imaging (TDI).

### BT2 Treatment

The compound BT2 (3,6-dichlorobenzo[b] thiophene-2-carboxylic acid) was obtained from the laboratory of Dr. David T. Chuang from UT Southwestern as a kind gift. Upon successful establishment of the HFpEF model based on the echocardiography data, BT2 or vehicle was administered at a dose of 40 mg/kg per day for 4 weeks as previously described 28. BT2 was dissolved in a pH 9.0 buffer solution containing 5% dimethyl sulfoxide, 10% Cremophor EL, and 85% 0.1 mol/L sodium bicarbonate. The BCKD complex activity in cardiac tissue, as well as the BCAA and BCAA downstream metabolite levels in both cardiac tissue and plasma, were measured as previously described 17.

### Radio Telemetry Monitoring of Blood Pressure

Systolic blood pressure (SBP), diastolic blood pressure (DBP), mean arterial pressure (MAP), pulse pressure (PP) and heart rate (HR) were measured using a radio telemetry system (Data Sciences International, St. Paul, MN, USA). The mice were anesthetized briefly with 1.5-2% isoflurane. The left common carotid artery was then isolated before the advancement of a catheter tip of a radio telemetry transmitter (PA-C10; Data Sciences International, St. Paul, MN, USA) into the thoracic aorta. The transducer was positioned along the left flank. An RPC-1 receiver system (Data Sciences International, St. Paul, MN, USA) was used to measure SBP, DBP, MAP, PP and HR. The data were subsequently collected and analyzed using Dataquest ART version 4.31 (DSI). Blood pressure monitoring was recorded every 5 min for two weeks after surgery.

### Histology and Masson's Trichrome Staining

The morphology was detected by hematoxylin and eosin (H&E) staining, and fibrosis was detected by Masson's trichrome staining. All stained samples were imaged with an EVOS M7000 Imaging System (Thermo Fisher Scientific, Pittsburgh, PA, USA).

### Glucose Tolerance Test

The mice were fasted for 6 hours and then injected intraperitoneally with D-glucose (Sigma) at a dose of 1.5 g/kg body weight. Blood glucose levels were detected in the tail vein by a portable glucometer at different timepoints (0, 15, 30, 60, and 120 min) after D-glucose injection.

### Exercise Exhaustion Test

After three days of acclimatization to the treadmill (Columbus Instruments), all mice underwent a warm-up period at a speed of 5 m/min for 4 min. Following this initial phase, the treadmill speed was incrementally increased every two min by 2 m/s until the mice reached exhaustion, defined as the point at which they were in contact with the electric-stimulus grid for 3 s. The running time and distance were recorded for each mouse during the test.

### BCAA and Downstream Metabolites Analysis

#### Sample preparation

The plasma and heart samples were stored at -80 °C until analysis. The plasma samples were thawed on ice, after which 10 µl was transferred to a new tube and extracted with 100 µl of acetonitrile: methanol (1:1, v/v) containing 5 µM internal standards. The samples were vortexed and centrifuged at 14,000 rpm for 10 min. The supernatants were then transferred to a new tube and dried in a vacuum concentrator at 4 °C. After the extracts were reconstituted with 50 µl of 5% acetonitrile in water, the mixture was vortexed for 2 min and centrifuged for 10 min at 14,000 rpm to remove the insoluble debris. The supernatants were transferred to glass vials for LC/MS injection. For heart tissue, 40 mg samples were homogenized with 400 µl of water at 4 °C. Then, 800 µl of acetonitrile: methanol (1:1, v/v) containing internal standards was added to 200 µl of tissue homogenate for metabolite extraction. The mixtures were vortexed, sonicated for 10 min at 4 °C in a water bath and incubated for 30 min at -80 °C to facilitate protein precipitation. The samples were centrifuged at 14,000 rpm for 10 min, and the supernatants were dried under a vacuum concentrator. Before LC/MS analysis, the dried samples were reconstituted with 100 µl of 5% acetonitrile in water, centrifuged for 10 min at 14,000 rpm and transferred to glass vials. The injection volume was set constant at 10 µl.

#### LC/MS analysis

The samples were run with a Waters BEH C18 column (150 × 4.6 mm, 2.5 μm, Waters, Milford, MA) on a Thermo UHPLC system coupled with a Q Exactive Plus mass spectrometer (QE MS, Thermo Scientific, San Jose, CA). The separation was performed using a gradient of solvent A (5 mM ammonium acetate and 0.1% ammonium hydroxide in water) and solvent B (methanol). The LC flow rate was 0.5 ml/min with an initial gradient of 2% B. The gradient program was as follows: 0-1 min 2% B, 1-5 min 20% B, 5-6 min 50% B, 6-8 min 90% B, and 8-10 min 90% B, followed by re-equilibration of the column to the initial run conditions (2% B) for 4.0 min. All the compounds were ionized in negative mode using electrospray ionization. The MS parameters are as follows: heater temperature, 350 °C; sheath gas, 55; auxiliary gas, 15; and spray voltage, 3.8 kV. The capillary temperature was set to 350 °C, and the S-lens was set to 50. The analysis was performed in parallel reaction monitor (PRM) mode with a resolution of 15,000 (at m/z 200), and the isolation window was set at 1.5 m/z. The raw data were collected using Thermo Xcalibur Software 4.1, and the data were processed using TraceFinder 4.1.

### Western Blot Analysis

After the mice were euthanized, 20 mg of the left ventricle was used for total protein extraction. Protein levels were measured and quantified using a BCA protein assay kit (PierceTM, Thermo Fisher Scientific). The proteins were resolved using sodium dodecyl sulfate-polyacrylamide gel electrophoresis (SDS-PAGE) and then transferred to polyvinylidene difluoride (PVDF) membranes (Millipore). The membranes were blocked with 5% milk and incubated with the following primary antibodies: anti-BCKDK, anti-BCKDHA, anti-p-BCKDHA (Ser 293), anti-p-AKT (Ser 473), anti-AKT, anti-p-AMPKα, anti-AMPKα, anti-p-P70S6K (Thr 389) and anti-P70S6K (Table S2). After washing, the membranes were incubated with an anti-rabbit IgG (H+L) HRP secondary antibody and an anti-mouse IgG (H+L) HRP secondary antibody (Table S2) for 1 h before imaging using Chemi-Doc (BioRad). Immunodetection of housekeeping proteins is routinely performed to detect differences in electrophoresis loading. Quantitative analysis of the images was performed using ImageJ software.

### RNA Extraction and Real-Time PCR

A total of 20 mg of the left ventricle was used for total RNA extraction with TRIzol Reagent (Thermo Fisher Scientific) following the manufacturer's instructions. One microgram of total RNA was reverse transcribed into cDNA using a reverse transcription kit (Takara Biotechnology, Japan). The expression of target genes was analyzed using CFX96 Touch Real-Time PCR system with SYBR Green (Bio-Rad). The relative expression levels of the target genes were detected according to the primers listed in Table S3.

### S-Nitrosylation Blot

S-Nitrosylation blot (Thermo Fisher) was conducted following the manufacturer's instructions. Protein was prepared at a concentration of 1 mg/ml in 100 µl of HENS buffer. For each analysis, 100 µg of protein was used to ensure equal amount across samples. Prior to precipitation with acetone, 2 µl of 1 M MMTS was added to the protein sample. The protein was then recovered in 100 µl of HENS buffer, labeled with the labeling reagent and sodium ascorbate, and allowed to react for 2 hours at room temperature prior to Western blot analysis.

### Pressure-Volume (PV) Loop Measurements

The PV loop was performed according to previously described methods 29. The mice were anesthetized via intraperitoneal injection of a mixture of 2% isoflurane and etomidate and then mechanically ventilated with a mixture of 2% isoflurane and 95% oxygen. The mice were placed on a heating pad, and the core body temperature was maintained at 37 °C. The left ventricle was exposed, and a specialized catheter with a diameter of 0.42 mm was inserted through the apex of the heart. The hemodynamic parameters were recorded and analyzed using LabChart 8.0 (AD Instruments).

### Statistical Analysis

The experimental data was analyzed using GraphPad Prism 8.3.0 and is presented as the means ± SEMs. The data distribution normality was determined using Shapiro-Wilk test for any sample size n > 6. For normally distributed data, parametric tests were used. Comparisons of two variables were tested using the Student's t-test. Comparisons among multiple variables were determined using one-way ANOVA with Tukey's or Fisher's post hoc test. For the analysis involving two categories of variables (time/treatment groups), two-way ANOVA followed by Tukey's test or LSD post hoc test was used as indicated specifically in the figure legend. *p* < 0.05 was considered statistically significant. The experimental schematics were designed using BioRender.

## Results

### Abnormal systemic BCAAs in HFpEF patients

**Figure 1:**
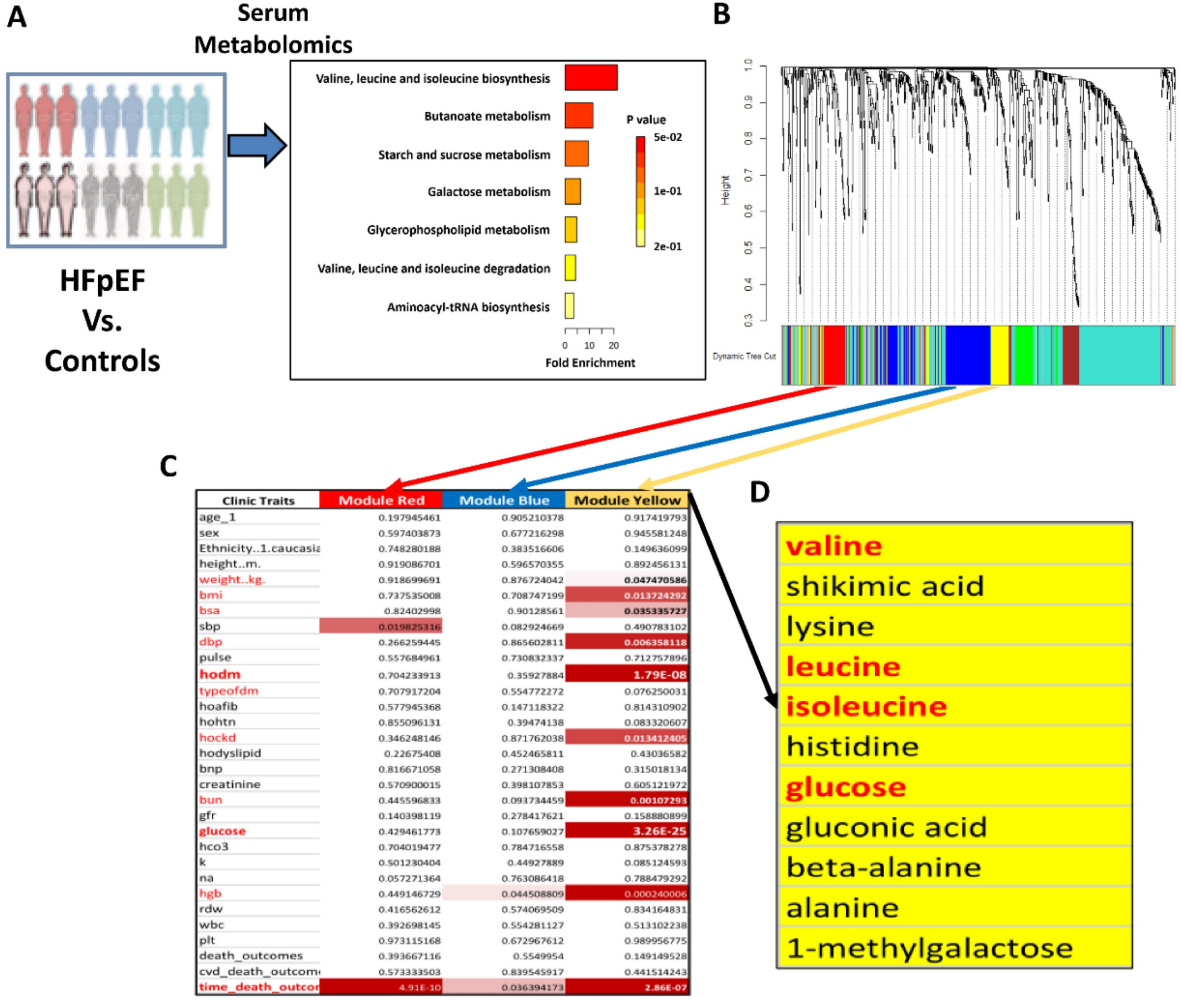
** BCAA is significantly increased in human with HFpEF. A,** Metabolic analysis results comparing HFpEF vs Control metabolite enrichments in KEGG pathways. X axis represents fold enrichment in HFpEF samples compared to control samples. Color reflects the p value. **B,** WGCNA dendrogram of 707 varying metabolites in our study population. **C,** P values showing the association of a subset of WGCNA modules to clinical traits. The yellow module shows significant enrichment for a number of traits. **D,** Major metabolites present within the yellow module. **Abbreviation:** Hoafib: Atrial fibrillation; BMI: Body Mass Index; BNP: Brain natriuretic peptide; BSA: Blood Serum Albumin; BUN: Blood urea nitrogen; CVD death: Cardiovascular death; DBP: Diastolic blood pressure; GRF: Glomerular filtration rate; Hgb: Hemoglobin; Hockd: Chronic kidney disease; Hodyslipid: Dyslipidemia; Hodm: Diabetes mellitus; Hohtn: Hypertention; HCO3: HCO-3; Plt: Platelet; SBP: Systolic blood pressure; K: Kalium; Na: Sodium; RDW: Red cell volume Distribution Width; WBC: White blood cell.

Metabolic disorders are known risk factors for HFpEF 30, 31, however, the underlying mechanism for the pathogenic contribution of metabolic impairments remains to be fully established. To identify potential metabolic pathways affected in HFpEF, we conducted comprehensive serum metabolomics profiling on a cohort of 255 deep-phenotyped patients with HFpEF alongside non-HFpEF controls 25. The demographic information and clinical characteristics of the study subjects are reported previously in detail 25. The mass spectrometry-based metabolomics profile covered nearly 200 primary metabolite species, including all amino acids and carbohydrate derivatives, 375 species of biogenic amines, and approximately 500 lipid species. We performed a systems-based analysis of these metabolites in association with the clinical outcome **(Figure 1A)**. Based on co-expression patterns among the cohorts, the metabolites were clustered into different modules using unsupervised clustering method (see Methods for details). Further correlation analysis was performed using principal component from each module against key clinical parameters, including metabolic indices (body mass index, fasting glucose level, and history of diabetes), cardiovascular indices (blood pressure, heart rate, hypertension, and pro-BNP level), kidney function indices (BUN, GFR, and CKD), and death outcomes **(Figure 1B)**. Among the different metabolite modules, the Module Yellow showed the most significant and highest number of correlations with detrimental clinical features, including early death, abnormal diastolic blood pressure, diabetes, obesity and kidney diseases** (Figure 1C)**. Further examination of the metabolite components within the Module Yellow revealed glucose- and gluconeogenesis-related metabolites, as well as a number of amino acids, including all members of the branched-chain amino acids (BCAAs): valine, leucine and isoleucine **(Figure 1D)**. This observation is in line with findings of an earlier report of metabolomics analysis from an independent cohort of HFpEF patients in which abnormal serum BCAA levels were also detected comparing with those of non-HFpEF patients 30, highlighting that abnormal BCAA level is a metabolic hallmark of HFpEF disease progression in human patients.

### BCAA catabolic pathway in HFpEF progression

**Figure 2:**
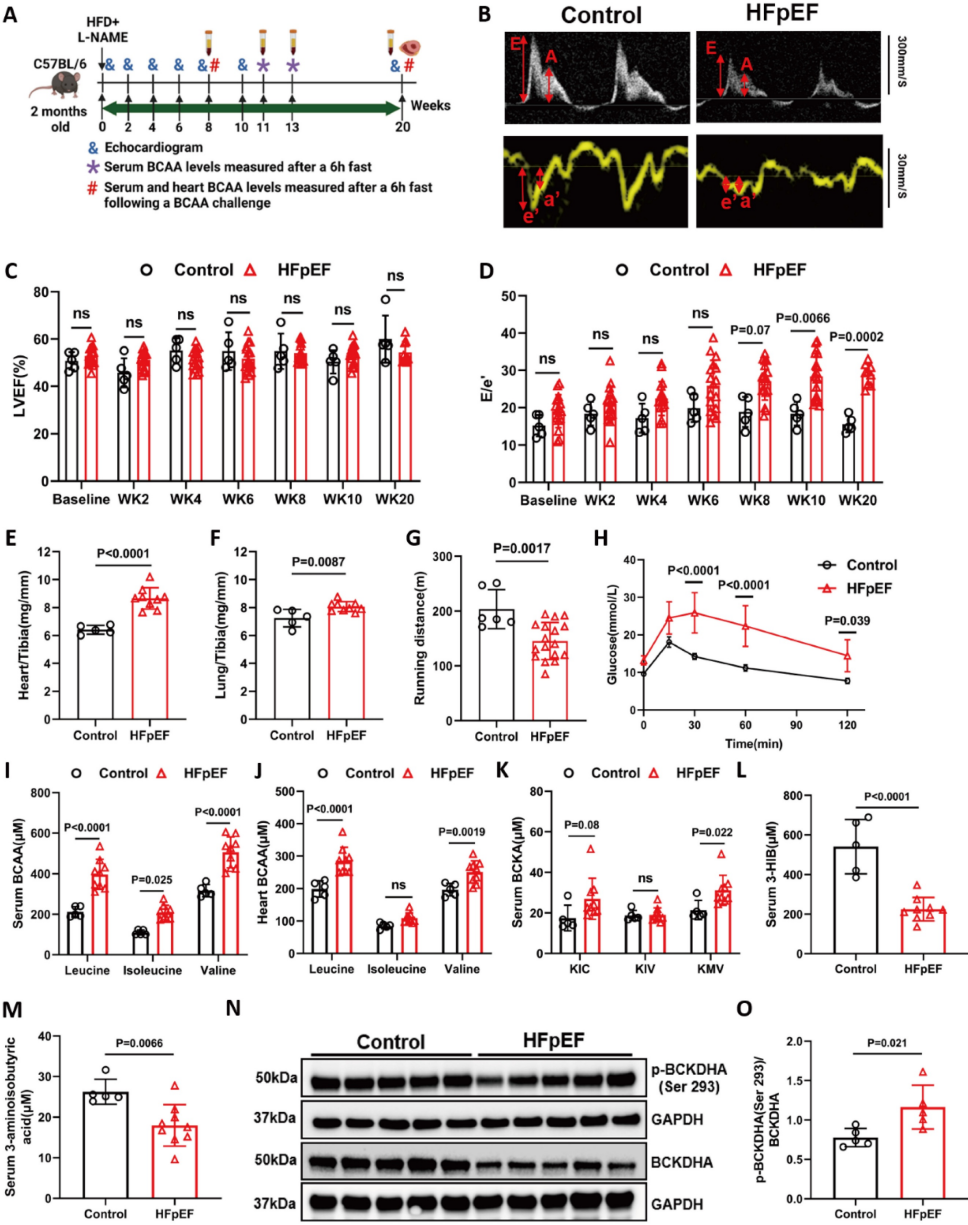
** BCAA is significantly increased in HFpEF mice. A,** Schematic view of experimental design. C57BL/6 mice were fed with HFD+L-NAME for 20 weeks with cardiac function and metabolic analyses conducted at indicated time points. **B,** Representative echocardiogram images (M-Mode) in control and mice fed with HFD+L-NAME for 14 weeks. **C,** Echocardiography analysis of left ventricle ejection fraction. n = 6-17. **D,** Mitral valve E/e' ratio. n = 6-17. **E,** Ratio of heart weight to tibia length (HW/TL). n = 5-9.** F,** Ratio of lung weight to tibia length (Lung/Tibia). n = 5-9. **G,** Treadmill analysis of running distance between the control and HFpEF mice. n = 5-9. **H,** Glucose tolerance test in Control and HFpEF groups. n = 5-9. **I,** Circulating BCAA levels at 20 weeks post HFpEF insult in Control and HFpEF mice. n = 5-9. **J,** Cardiac BCAA levels at 20 weeks post HFpEF insult. n = 5-9.** K,** Circulating BCKA levels at 20 weeks post HFpEF insult. n = 5-9. **L-M,** Circulating 3-HIB (L) and 3-aminoisobutyric acid (M) levels at 20 weeks post HFpEF insult. n = 5-9. **N-O,** Representative immunoblot (N) and quantification (O) of heart p-BCKDHA (Ser 293), BCKDHA and GAPDH protein. n = 5 per group. Two-way ANOVA followed by Turkey's test was used in C-D and One-way ANOVA followed by Turkey's test was used in I, J, K and L. Student t-test was used in E, F, G, M and O. Repetitive t-test was used in H.

To further establish the correlation between BCAA catabolic changes and HFpEF, we investigated the changes in BCAA levels throughout the progression of HFpEF using a well-established preclinical mouse model 27. Adult male C57BL/6 mice were administered 0.5 mg/ml L-NAME in their drinking water and fed a 60% HFD for up to 20 weeks. Cardiac function was assessed by echocardiography, and blood samples were collected at different time points as indicated** (Figure 2A-B)**. As reported earlier, these mice developed cardiac pathologies characteristic of HFpEF over time, including preserved ejection fraction and fraction shortening **(Figure 2C and S1A)**, elevated E/e' ratio **(Figure 2D)** and prolonged isovolumic relaxation time (IVRT) as early as 8 weeks after L-NAME/HFD treatment** (Figure S1B)**, along with an increasing trend in the E/A ratio **(Figure S1C)**. When assessed at week 20 post induction of HFpEF, these mice also exhibited significant cardiac hypertrophy, pulmonary congestion and exercise intolerance **(Figure 2E-G)**, as well as impaired systemic glucose clearance **(Figure 2H)**. Cardiac remodeling was further demonstrated by extensive cardiac fibrosis based on histology and Masson's trichrome staining **(Figure S2)**. At the molecular level, the HFpEF hearts exhibited increased expression of genes related to cardiac hypertrophy, inflammation and fibrosis, including B-type natriuretic peptide (BNP), C-reactive protein (CRP) and Collagen type I alpha 1 chain (Col1α1) **(Figure S3A-C)**, as well as significantly elevated global protein S-nitrosylation **(Figure S3D-E)**. These data indicate that, in agreement with previous publications, early signs of diastolic impairment developed after a minimum of 10 weeks of L-NAME/HFD treatment, which progressed into profound diastolic dysfunction and pathological remodeling in the heart at week 20.

To cover the period of critical transition during the onset and progression of HFpEF, we then measured the levels of BCAA and BCKA in plasma from the same experimental cohorts at three time points, including weeks 8, 11 and 13 after HFpEF induction. Notably, fasting BCAA/BCKA levels were significantly elevated at week 13 of HFpEF induction but not prior to this time point **(Figure S4A-F)**. When the experiment was terminated at week 20 post HFpEF induction, the BCAA catabolic defect was evident, as both serum and intra-tissue BCAA and BCKA levels were significantly elevated in response to BCAA challenge, whereas downstream BCAA metabolites were markedly lower than those in non-HFpEF control mice **(Figure 2I-M)**. These data suggest that BCAA catabolic defects develop with the onset and progression of HFpEF.

To elucidate the mechanism of BCAA metabolic defect in HFpEF, we determined the levels of key proteins/enzymes involved in BCAA catabolism in the heart tissue of HFpEF mice at week 20 post HFpEF induction. Quantitative RT-PCR analysis showed a significant increase in the expression of *Bckdha* and *Bckdk* mRNA **(Figure S4G)**, whereas Western blot analysis revealed a significant decrease in BCKDHA and PP2Cm protein expression but elevated BCKDHA phosphorylation in the HFpEF hearts compared with that in control hearts **(Figure 2N-O and S4H-I)**. Since BCKDHA phosphorylation represses BCKDH activity, both molecular and metabolic analyses indicated a state of impaired BCAA catabolic flux in HFpEF hearts at the step of rate-limiting BCKDH activity **(Figure S4J)**.

### Global BCAA catabolic activation attenuated cardiac dysfunction and remodeling

While BCAA catabolic defect appears to be significantly associated with the onset and progression of HFpEF, the functional impact has never been directly demonstrated. To generate a mouse model with increased global BCAA catabolic flux, we crossed *bckdk^flox/flox^* mice 26 with a transgenic line expressing tamoxifen inducible Cre (ERT2-Cre) under a human ubiquitin C promoter (BCKDK-uKO) **(Figure S5A)**. Compared with tamoxifen-treated control mice carrying only the bckdk^flox/flox^ allele, the resulting BCKDK-uKO mice presented a marked reduction in BCKDK mRNA in metabolic organs after two weeks of a tamoxifen-containing diet **(Figure S5B)**. As expected, the phosphorylation of BCKDHA at Ser 293 was dramatically decreased in BCKDK-uKO heart and other metabolic tissues **(Figure S5C-E)**. BCKDK-uKO mice exhibited gross morphology and metabolic phenotypes similar to those of the control group, including fat mass, lean mass, body weight and glucose tolerance (**Figure S5F-I**). We assessed cardiac function using echocardiography at 20-24 weeks of age for BCKDK-uKO and control mice, and found that BCKDK-uKO mice exhibited similar cardiac function to control mice, including LVEF, FS, IVRT, E/e' and E/A ratio (**Figure S5J-N**). The heart failure markers Atrial natriuretic peptide (ANP) and BNP, along with the fibrosis markers Col1a1 and Col3a1, showed no significant differences between the two groups (**Figure S5O-R**). Cardiac fibrosis and structure in BCKDK-uKO mice are similar to those in control mice (**Figure S5S-T**). The serum BCAA/BCKA ratio was markedly lower than that in control heart tissue **(Figure S5U-V)**. These findings suggest that global Bckdk gene inactivation does not alter metabolic phenotypes or cardiac function, yet increases BCAA flux under baseline condition.

**Figure 3:**
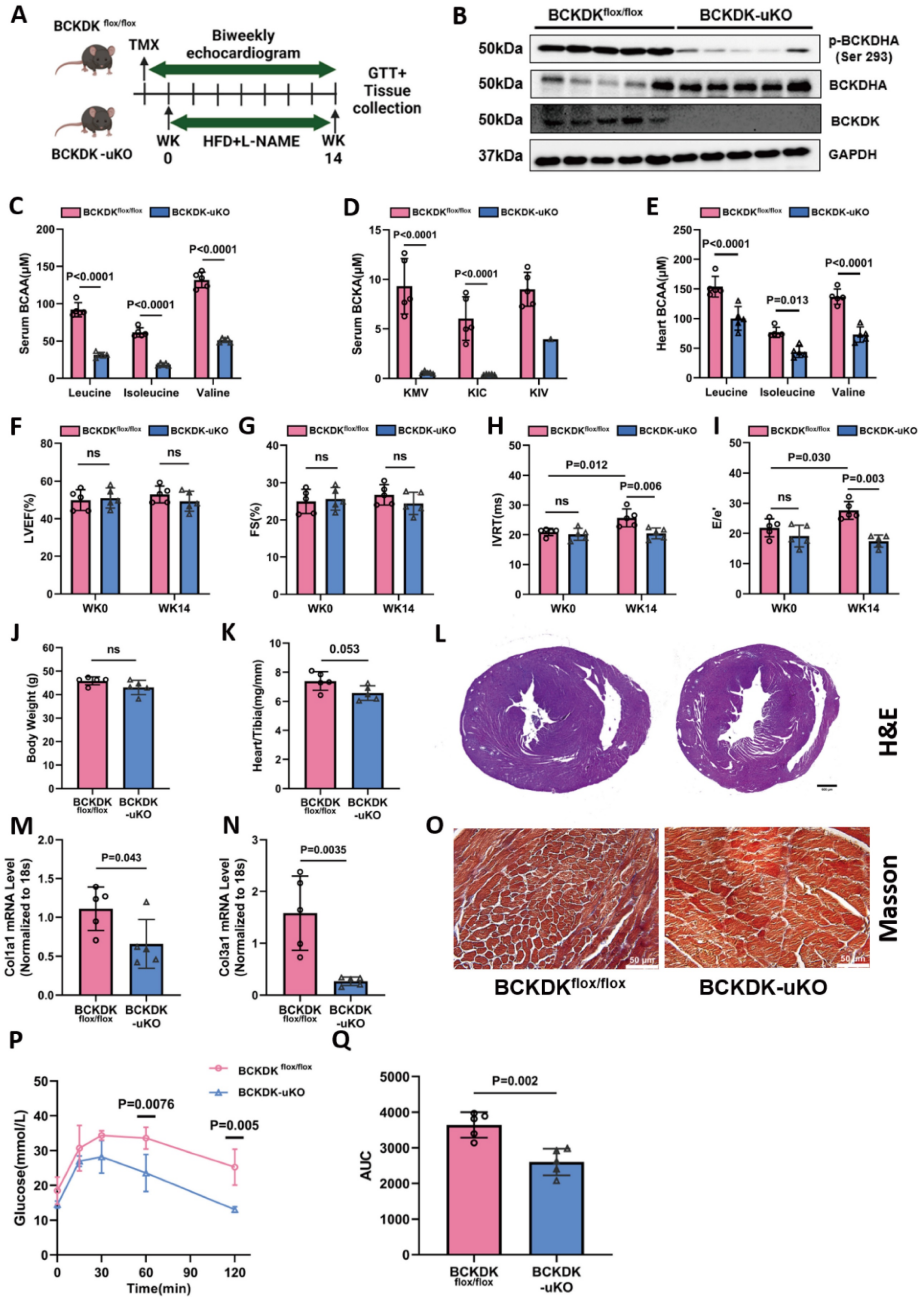
** Global BCAA catabolism activation prevents cardiac dysfunction in Two-Hit HFpEF model. A**, Schematic view of experimental design. BCKDK^flox/flox^ and BCKDK-uKO mice were fed with tamoxifen diet for 2 weeks before treated with HFD+L-NAME for 14 weeks. Cardiac function and metabolic assessment were performed at indicated time points. **B**, Western blot analysis of p-BCKDHA (Ser 293), BCKDHA, BCKDK and GAPDH protein levels in BCKDK^flox/flox^ and BCKDK-uKO mice. n = 5. **C-D**, Serum BCAA (C) and BCKA (D) levels in BCKDK^flox/flox^ and BCKDK-uKO mice. n = 5. **E**, Cardiac BCAA levels in BCKDK^flox/flox^ and BCKDK-uKO mice. n = 5. **F-I**, Echocardiography analysis of left ventricle ejection fraction (F), fraction shortening (G), IVRT (H) and mitral valve E/e' ratio (I) in BCKDK^flox/flox^ and BCKDK-uKO mice. n = 5. **J**, Body weight of BCKDK^flox/flox^ and BCKDK-uKO mice. n = 5. **K**, Ratio of heart weight to tibia length (HW/TL) in BCKDK^flox/flox^ and BCKDK-uKO mice. n = 5. **L**, H&E staining for cardiac section in BCKDK^flox/flox^ and BCKDK-uKO mice post HFpEF insult. Scale bars, 600μm (H&E). **M-N**, Real-Time PCR analysis of mRNA expression of Col1a1 (M) and Col3a1 (N) in BCKDK^flox/flox^ and BCKDK-uKO mice post HFpEF stimulation. n = 5. **O**, Masson's trichrome staining for left ventricle section in BCKDK^flox/flox^ and BCKDK-uKO mice post HFpEF insult. Scale bars, 50μm (Masson). **P-Q**, Glucose tolerance test (P) and area under curve (Q) in BCKDK^flox/flox^ and BCKDK-uKO mice post HFpEF insult. n = 5. One-way ANOVA followed by Turkey's test was used for C-I. Student t-test was used in J-K, M-N, Q. Repetitive t-test was used for P.

Adult male BCKDK-uKO and control mice were subsequently treated with L-NAME (0.5 mg/ml) and a 60% HFD for 14 weeks (**Figure 3A**). Consistent with baseline findings, BCKDK deficiency markedly decreased both BCKDK and phosphorylated BCKDHA protein levels compared to control mice (**Figure 3B**), and the serum BCAA/BCKA levels were significantly lower than that in control mice (**Figure 3C-D**). The intracardiac tissue BCAA levels were markedly lower (**Figure 3E**), whereas the cardiac BCKA levels showed a lower trend in the BCKDK-uKO mice than in the control genotype (**Figure S6A**). We further detected the BCKA derivative metabolites, including 2-hydroxy-3-methylvaleric acid and 2-hydroxyisocaproate (derived from KMV and KIC, respectively) and showed that they exhibited the same trend of reduction, providing the indirect support for the reduced BCKA levels in the BCKDK-uKO mouse hearts (**Figure S6B-C**), validated again that global inactivation of the *Bckdk* gene leads to increased BCAA flux. Echocardiography demonstrated that the BCKDK-uKO mice showed a marked improvement in left ventricular (LV) diastolic function, as evidenced by a significant reduction in the E/e' ratio and IVRT, whereas the LVEF and FS remained unaffected post HFpEF stimulation **(Figures 3F-I)**. In addition, *Bckdk* inactivation did not affect total body weight but significantly reduced cardiac hypertrophy, as demonstrated by decreased heart weights **(Figure 3J-L)**. The BCKDK-uKO mice showed a significant reduction in the expression of the Col1α1 and Col3α1 genes, as well as a decrease in cardiac fibrosis, as determined by Masson's trichrome staining** (Figure 3M-O)**. Importantly, the BCKDK-uKO mice displayed improved glucose clearance capacity **(Figure 3P-Q)**. These results clearly indicate that enhancing BCAA catabolic flux can significantly ameliorate cardiac diastolic dysfunction, cardiac remodeling and metabolic disorder under HFpEF insult.

### Enhancing BCAA catabolic activity attenuates cardiac insulin resistance and nitrosative injury

**Figure 4:**
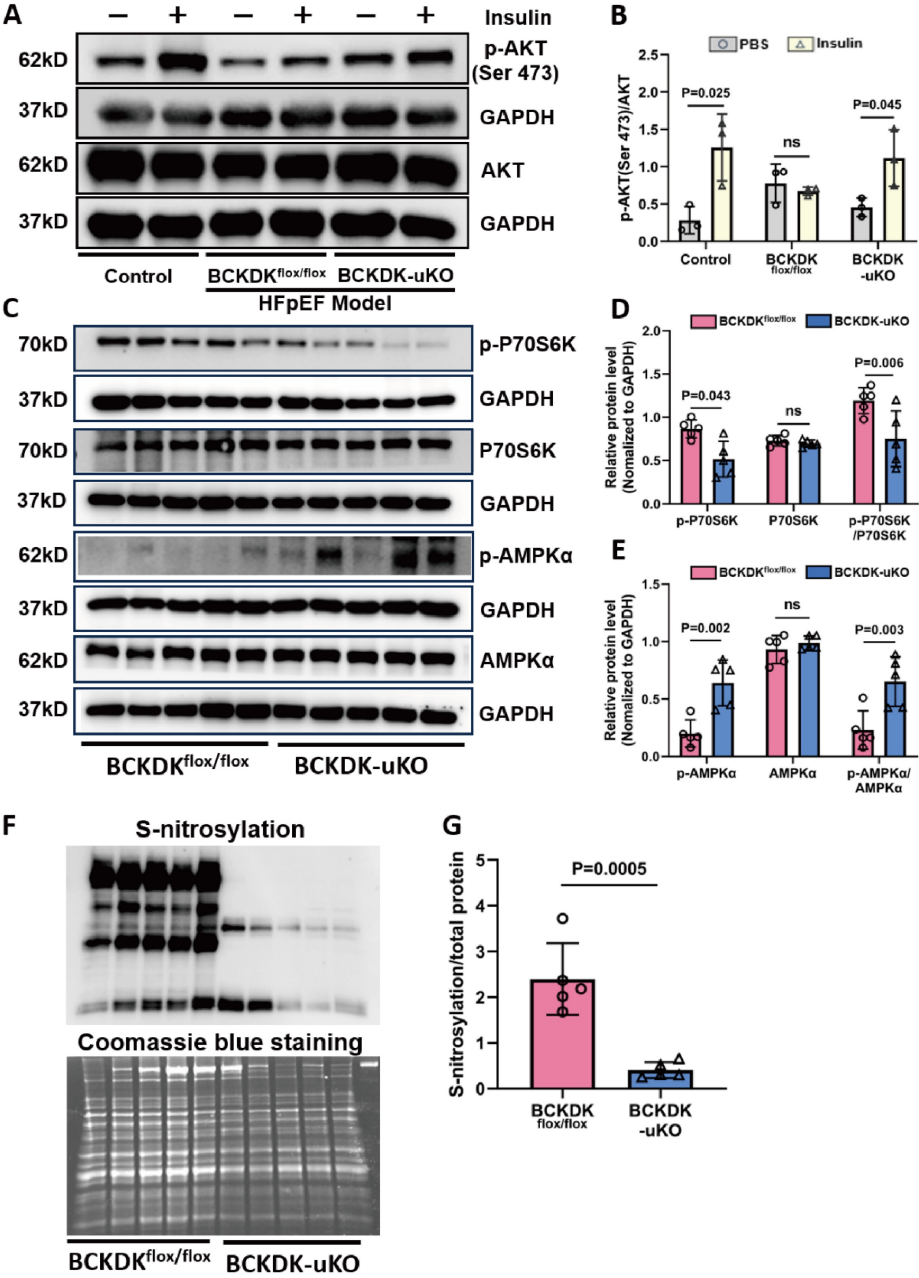
** Global BCAA catabolism activation alters insulin signaling pathway in heart. A-B,** Western blot analysis (A) and quantification (B) of p-AKT (Ser 473), AKT and GAPDH in left ventricle tissues from different groups: Control without HFpEF insults, BCKDK^flox/flox^ and BCKDK-uKO following HFpEF insults, under insulin or PBS (control) treatment. n = 3. **C-E,** Western blot analysis (C) and quantification (D-E) of p-P70S6K, total P70S6K, p-AMPKα, total AMPKα and their corresponding GAPDH as loading control in BCKDK^flox/flox^ and BCKDK-uKO mice after HFpEF insults. n = 5. **F-G,** Western blot analysis (F) and quantification (G) of s-nitrosylation status in BCKDK^flox/flox^ and BCKDK-uKO mice following HFpEF insults. n = 5. One-way ANOVA followed by Tukey's test was used for B, D and E. Student t-test was used for G.

BCAA catabolic defect is found to be a significant causal factor of insulin resistance associated with obesity 32-34, and diabetes/obesity are also major metabolic risk factors for HFpEF 35. To assess the impact of BCAA catabolism on cardiac insulin signaling, we measured key signaling pathways downstream of the insulin receptor. As shown in **(Figure 4A-B)**, BCKDK-uKO hearts presented significantly greater insulin-induced AKT phosphorylation in cardiac tissue after 14 weeks of L-NAME/HFD treatment than control hearts. Meanwhile, the BCKDK-uKO mice presented a significant reduction in phosphorylated P70S6K and an increase in phosphorylated AMPKα in the heart** (Figure 4C-E)**, supporting a state of attenuated mTOR activity and restored metabolic signaling. Additionally, we observed a remarkable reduction in S-nitrosative stress level in BCKDK-uKO hearts compared with those in control hearts under HFpEF stress **(Figure 4F-G)**. These results support the notion that enhancing BCAA catabolic flux ameliorated the key molecular features associated with HFpEF involving insulin signaling and NOS signaling defect, as well as nitrosative stress level.

### BCKDK inactivation does not affect blood pressure

**Figure 5:**
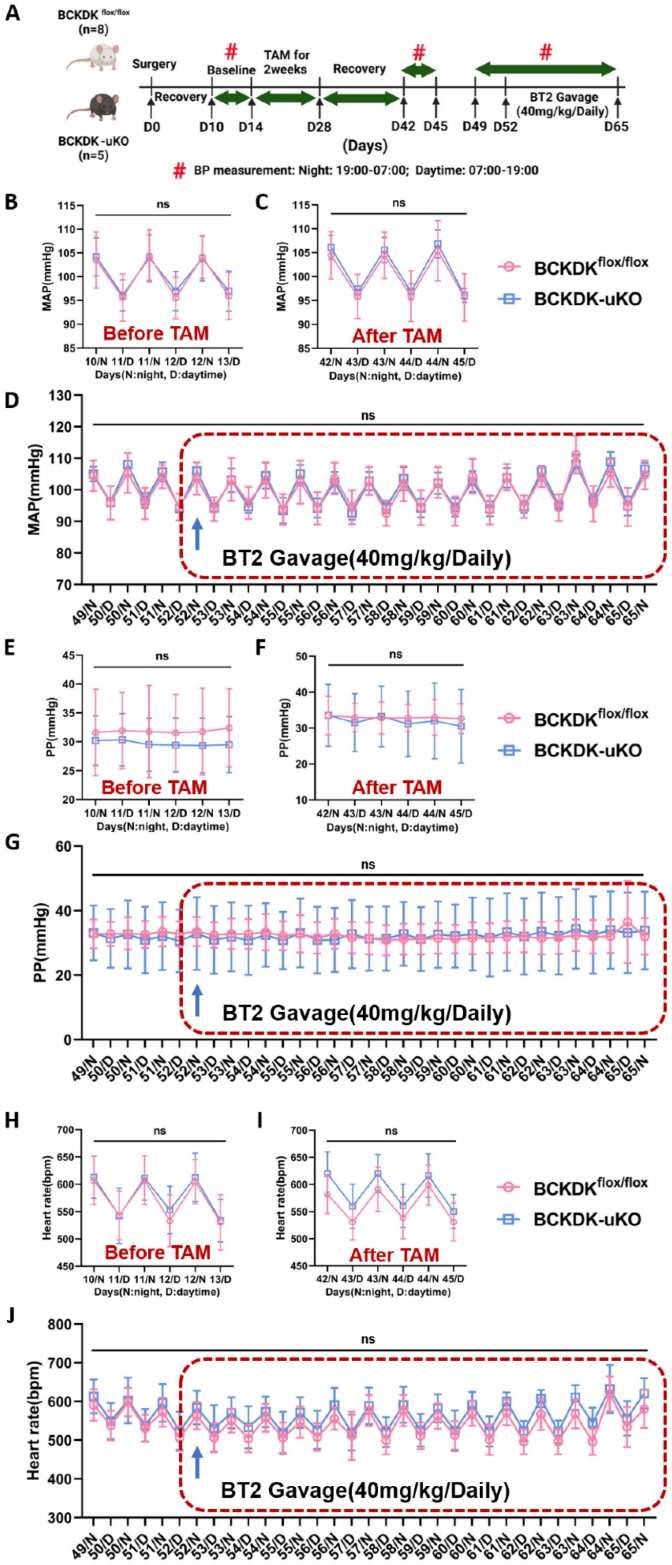
** Targeting BCKDK to accelerate BCAA catabolism has no impact on blood pressure. A,** Schematic view of experimental design. BCKDK^flox/flox^ and BCKDK-uKO mice were fed with tamoxifen and treated with BT2 or vehicle, and blood pressure was measured using telemetry, Average daily pressure over course of telemetry experiment from light cycle (7:00-19:00) and dark cycle (19:00-7:00). **B-C,** Mean arterial pressure measured via telemetry in BCKDK^flox/flox^ and BCKDK-uKO mice before (B) and after (C) tamoxifen treatment. n = 4-8. **D,** Mean arterial pressure measured via telemetry in BCKDK^flox/flox^ and BCKDK-uKO mice before and after BT2 treatment. n = 4-8. **E-F,** Pulse pressure measured via telemetry in BCKDK^flox/flox^ and BCKDK-uKO mice before (E) and after (F) tamoxifen treatment. n = 4-8. **G,** Pulse pressure measured via telemetry in BCKDK^flox/flox^ and BCKDK-uKO mice before and after BT2 treatment. n = 4-8. **H-I,** Heart rate measured via telemetry in BCKDK^flox/flox^ and BCKDK-uKO mice before (H) and after (I) tamoxifen treatment. n = 4-8. **J,** Heart rate measured via telemetry in BCKDK^flox/flox^ and BCKDK-uKO mice before and after BT2 treatment. n = 4-8. BT2 was administered via oral gavage once a day at 18:00. Two-way ANOVA followed by Turkey's test was used for B-J.

Hypertension and metabolic disorders are two major contributing factors to the pathogenesis of HFpEF, as demonstrated in clinical and preclinical models 36, 37. A previous study reported that systemic BCAA catabolic activity is associated with increased blood pressure 38, raising the question of whether enhancing BCAA catabolic activity could provide protective effects against HFpEF by reducing blood pressure. To test this possibility, we continuously measured systemic blood pressure in conscious mice using implanted telemetric probes as described in the Methods section **(Figure 5A and S7A)**. A comparison of blood pressure before and after tamoxifen treatment in BCKDK-uKO and BCKDK^flox/flox^ controls revealed no differences in systolic or diastolic blood pressure, mean arterial pressure and pulse pressure, or heart rate during each circadian cycle **(Figure S7B-C and S7E-F and 5B-C and 5E-F and 5H-I)**. We further evaluated the effect of the BCKDK inhibitor BT2 on blood pressure in both BCKDK-uKO and BCKDK^flox/flox^ control mice, and no changes in MAP, PP, HR, SBP and DBP were observed following 2 weeks of BT2 treatment** (Figure 5D and 5G and 5J and S7D and S7G)**. These results suggest that the amelioration of HFpEF pathogenesis by targeting BCKDK is likely due to restored BCAA catabolic flux rather than lowered blood pressure.

### Pharmacological inhibition of BCKDK boosts BCAA catabolism in HFpEF

**Figure 6:**
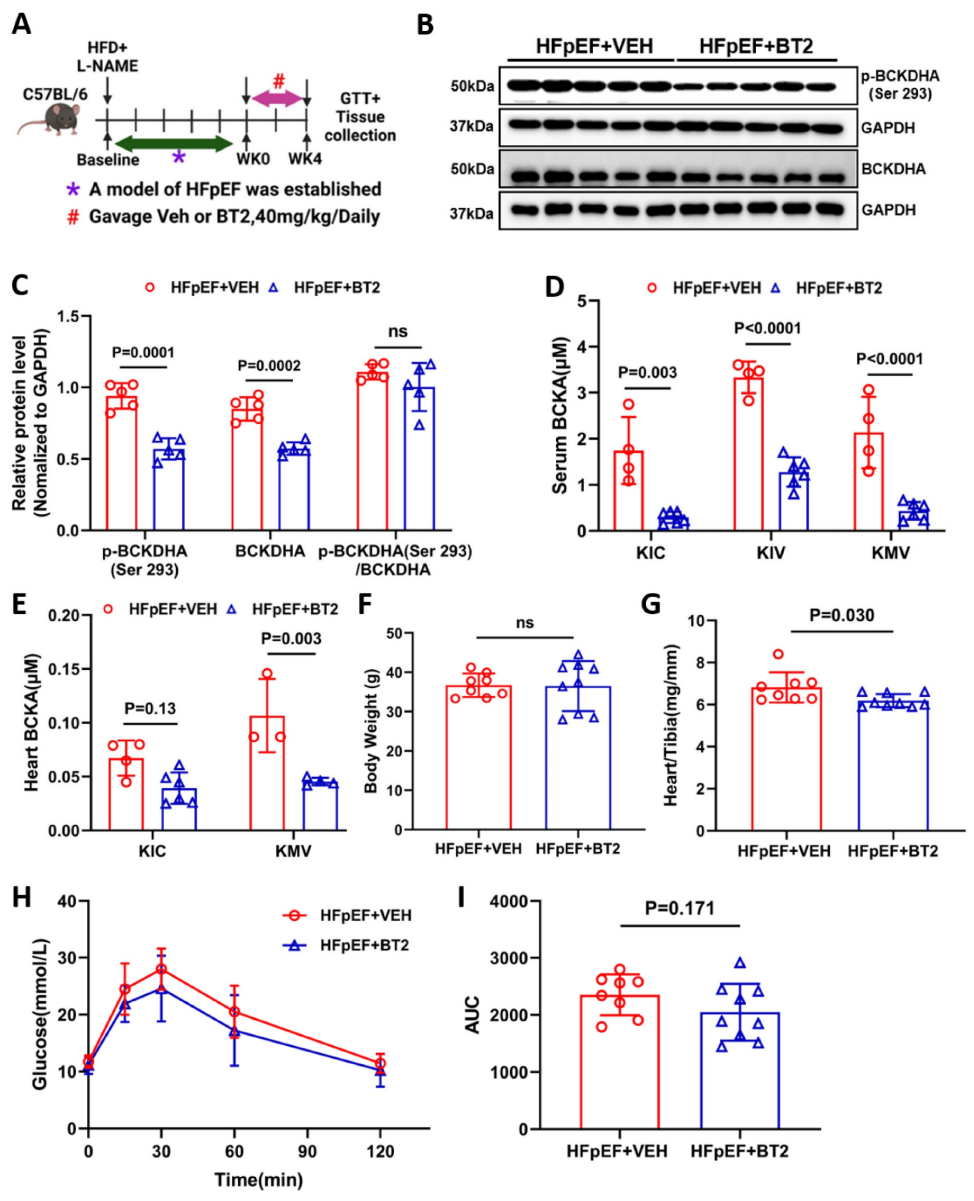
** Activation BCAA catabolism using BT2 accelerates BCAA catabolism. A,** Schematic view of experimental design. C57BL/6 mice were fed with HFD+L-NAME for 10 weeks before treated with BT2 or vehicle for 4 weeks, cardiac and metabolic analysis was performed as indicated. **B-C,** Representative immunoblot (B) and quantification (C) of heart p-BCKDHA (Ser 293), total BCKDHA and GAPDH protein levels in vehicle and BT2 treated HFpEF groups. n = 5. **D,** Circulating BCKA levels in BT2 or vehicle treated HFpEF groups. n = 4-6.** E,** Cardiac BCKA levels in BT2 or vehicle treated HFpEF groups. n = 4-6. **F,** Body weight of BT2 or vehicle treated HFpEF groups. n = 8-9. **G,** Ratio of heart weight to tibia length (HW/TL). n = 8-9. **H-I,** Glucose tolerance test (H) and area under curve (I) in BT2 or vehicle treated HFpEF groups. n = 8-9. One-way ANOVA followed by Turkey's test was used for C-E. Student t-test was used for F-G and I. Repetitive t-test was used for H.

While genetic inactivation of BCKDK had a significant beneficial effect on the pathogenic progression of HFpEF in mice, the therapeutic efficacy of BCKDK inhibition in established HFpEF is unknown. Therefore, we investigated whether pharmacological inhibition of BCKDK with a highly specific inhibitor, BT2 39, 40, could rebalance BCAA catabolism and improve cardiac function in HFpEF mice. In this study, 2-month-old male C57BL/6 mice were fed a HFD and L-NAME for 10 weeks and then randomized to receive either BT2 (40 mg/kg/daily oral gavage) for 4 weeks as the treatment group or vehicle as the placebo group **(Figure 6A)**. Mice treated with BT2 effectively inhibited BCKDK activity, as evidenced by decreased level of phosphorylated BCKDHA **(Figure 6B-C)**. Furthermore, mass-spectrum analysis showed a significant decrease in BCKAs and their downstream metabolites-3-hydroxyisobutyrate (3-HIB) and 3-aminoisobutyric acid- levels in both serum and myocardial tissue in the HFpEF mice after BT2 treatment comparing with the vehicle treated controls **(Figure 6D-E and S8A-D)**. Similar to the observation in the BCKDK-uKO mice, BT2 treatment significantly reduced cardiac hypertrophy without affecting body weight under HFpEF insult **(Figure 6F-G)**. Compared with the vehicle control, BT2 treatment also tended to improve glucose clearance **(Figure 6H-I)**. Taken together, these results indicate that BT2 treatment can reset BCAA catabolism and improve metabolic remodeling in HFpEF.

### BT2 therapy reverses cardiac diastolic dysfunction in HFpEF

**Figure 7:**
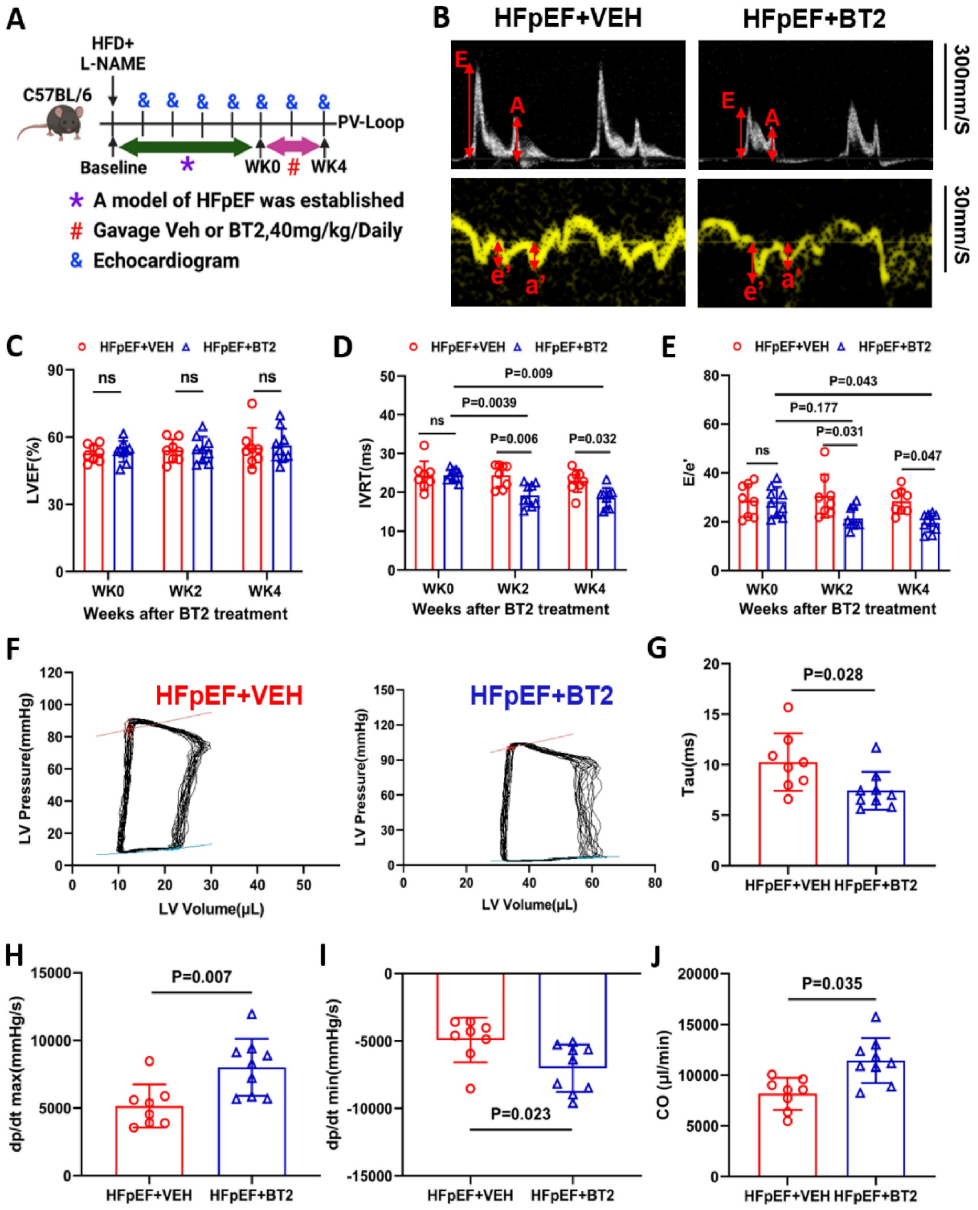
** Pharmacological activation of BCAA catabolism prevents cardiac dysfunction. A,** Schematic view of experimental design. C57BL/6 mice were fed with HFD+L-NAME for 10 weeks before treated with BT2 or vehicle for 4 weeks, cardiac and metabolic analysis was performed as indicated. **B**, Representative echocardiogram images of mitral valve E/A and doppler e'/a' in HFpEF mice after 4 weeks of vehicle or BT2 treatment. **C-E,** Echocardiography analysis of LVEF (C), Isovolumic relaxation time IVRT (D) and mitral valve E/e' ratio (E) at baseline and after 4 weeks of vehicle/BT2 treatment. n = 8-9. **F,** Representative Pressure Volume Loops in HFpEF mice post vehicle or BT2 treatment for 4 weeks. **G,** Catheter analysis of relaxation time constant (Tau) in HFpEF mice after 4 weeks of vehicle and BT2 treatment. n = 8-9. **H,** Catheter analysis of dp/dt max in HFpEF mice after 4 weeks of vehicle and BT2 treatment. n = 8-9. **I,** Catheter analysis of dp/dt min in HFpEF mice after 4 weeks of vehicle and BT2 treatment. n = 8-9. **J,** Catheter analysis of cardiac output (CO) in HFpEF mice after 4 weeks of BT2 treatment. n = 8-9. Two-way ANOVA followed by Turkey's test was used for C-E. Student t-test was used for G-J.

Cardiac function was measured prior to and after BT2 therapy over time **(Figure 7A)**. Notably, the administration of BT2 reversed the cardiac diastolic dysfunction induced by L-NAME/HFD. This was demonstrated by a reduced E/e' ratio and IVRT 4 weeks after BT2 treatment compared with those of the same animal cohort prior to BT2 treatment at week 10 of HFpEF induction or the placebo control group at any time point, without affecting the left ventricular ejection fraction and fraction shortening **(Figure 7B-E and S9A)**. To further confirm the protective effect of BT2 on cardiac function, invasive hemodynamic measurements were performed to obtain pressure-volume loop at the end of the treatment protocol. As shown in **Figure 7F-G**, BT2 therapy significantly improved cardiac function, as evidenced by the decrease in tau in the BT2-treated group compared with that of the vehicle-treated control group. In addition, BT2 treatment significantly increased cardiac output (CO) and improved systolic and diastolic performance, as demonstrated by the higher levels of dp/dt_max_ and dp/dt_min_ in the treatment group than in the vehicle-treated controls **(Figure 7H-J)**. Additionally, we observed a significant reduction in S-nitrosative stress level in the HFpEF hearts treated with BT2 compared to the vehicle-treated group **(Figure S9B-C)**. Overall, these data demonstrated that BT2 therapy significantly reversed cardiac dysfunction in established HFpEF.

## Discussion

In this study, metabolomics analysis of both a patient cohort and a mouse model of HFpEF revealed that impaired BCAA catabolism is a key pathogenic feature of HFpEF. Based on histological and functional analyses in the HFpEF mouse model, inhibition of BCKDK genetically or pharmacologically demonstrates potent therapeutic efficacy against metabolic and fibrotic remodeling, leading to improved diastolic function in HFpEF. Mechanistically, this improvement is linked to enhanced cardiac insulin signaling and reduced NOS injury, with no observed effect on blood pressure. These findings support the notion that impaired BCAA catabolism is a major pathogenic feature of HFpEF and a promising therapeutic target for this disease.

HFpEF represents a significant unmet medical need and is often associated with notable metabolic dysfunctions, such as insulin resistance and lipotoxicity 41. In HFpEF, the heart is characterized by energetic defects with an increased cellular pool of FA-derived acetyl-CoA that promotes protein acetylation, reduces glucose metabolism, and enhances fatty acid oxidation (FAO) 42. Moreover, amino acids, which serve as both important nutrients and potent signaling molecules, can also exert significant effects on glucose and lipid metabolism to maintain cardiac function 43, 44. The BCAAs—isoleucine, leucine, and valine—are essential in protein synthesis and play a key role in regulating metabolic health 45-47. A previous study revealed that impaired BCAA catabolism contributes to the progression of HFrEF 17, 21. A clinical study from the PRESERVED-HF trial also confirmed that abnormal serum BCAA levels were associated with HFpEF disease severity 48. In this study, we analyzed BCAA levels and related metabolites at different time points during the progression of HFpEF in a mouse model. Both the serum and cardiac levels of BCAAs and BCKAs are significantly increased, whereas downstream metabolites are decreased longitudinally in association with the progression of HFpEF phenotypes. Consistent with the changes in BCAA metabolism, there was a significant increase in the ratio of p-BCKDHA (Ser 293) to BCKDHA, indicating an impaired BCAA catabolic pathway. Therefore, both clinical and preclinical studies suggest that BCAA catabolic defects are associated with HFpEF progression and may serve as a target for therapeutic intervention.

Previous studies have demonstrated that systemic activation of BCAA oxidation with BT2 can improve HFrEF induced by pressure overload or ischemic injury 28, 49. BT2 is recognized as a highly specific BCKDK inhibitor, which underlies its therapeutic effect on insulin resistance and heart failure 50, 51. However, there is evidence suggesting that BT2 may have potential "off-target" effects, such as promoting tryptophan catabolism 39. In our current study, we employed global *Bckdk* knockout mice to clarify whether the effect of BT2 on HFpEF pathogenesis is on-target. Indeed, we found that genetic knockout of *Bckdk* can significantly improve diastolic function, heart weight, and insulin resistance and reduce cardiac fibrosis, which is associated with a significant increase in BCAA oxidation. These findings indicate that the cardioprotective effect of BT2 in HFpEF likely also results from its inhibition of BCKDK activity and subsequent enhancement of BCAA catabolism.

Mechanistically, we found that increasing the oxidation of BCAAs provides cardioprotective benefits against HFpEF associated with improved insulin resistance without affecting blood pressure. Clinical studies have shown that diabetes is linked to a worse clinical prognosis and a greater risk of adverse cardiovascular events in HFpEF patients 52, 53. Metabolomics studies in HFrEF have indicated that higher cardiac levels of BCAAs are associated with increased cardiac insulin resistance and reduced cardiac function 54, 55. Our previous study demonstrated that reducing insulin resistance with a glucagon receptor antagonist improves cardiac diastolic function across different HFpEF models 56. Our results from the current study also indicate that genetic inhibition of *Bckdk* leads to increased phosphorylation of AKT serine/threonine kinase 1 in response to insulin stimulation. Moreover, we observed a notable reduction in nitrosative stress levels in global *Bckdk* knockout mice with HFpEF, indicating the molecular downstream effects of BCAA catabolic activity on insulin signaling and NOS regulation. Hypertension is a notable comorbidity of HFpEF 57. In this setting, dapagliflozin has demonstrated benefits by improving left ventricular remodeling and reducing aortic sympathetic tone in a pig model of HFpEF 58. Pharmacological activation of BCAA oxidation has been shown to lower blood pressure in a previous report 38. However, when we used a sensitive implanted telemetry method, we did not observe any notable changes in systemic blood pressure caused by either genetic or pharmacological inhibition of BCKDK activity in mice. Therefore, insulin sensitivity and NOS signaling are likely crucial downstream mechanisms underlying the pathogenic and cardioprotective effects of BCAA catabolism. As numerous signaling effects of BCAAs, including mTOR and autophagy, have been reported, more studies are needed to establish the specific molecular processes linking BCAA catabolism and HFpEF pathologies in the heart.

In this study, both pharmacological and genetic inhibition of BCKDK activity affected global BCAA catabolic activities across different organs. In our previous studies, cardiomyocyte-specific inhibition of *bckdk* failed to confer cardio-protection against HFrEF 26, supporting the potential contribution of the systemic effect of BCAA catabolism on HFpEF pathogenesis. While the focus of the current study was systemic modulation of BCAA, the role of BCAA catabolism in cardiomyocytes or other organs, such as the liver, skeletal muscle, and adipose tissue, still requires further investigation. Even in the heart, the crucial mechanism underlying the effects of BCAA oxidation in various cell types in addition to myocytes, such as fibroblasts, endothelial cells, and macrophages, still remains to be clarified. Importantly, systemic treatment with BCKDK inhibitors is currently undergoing clinical study for HFpEF 59, 60. The present study is the first proof-of-concept demonstration of targeted BCAA catabolism therapy and a model system for future studies focusing on the mechanism of action.

## Supplementary Material

Supplementary figures and tables.
