# Supplementary material for: BCAA catabolism targeted therapy for heart failure with preserved ejection fraction
Source: Theranostics. 2025 May 24;15(13):6257–73. doi: 10.7150/thno.105894 (PMC12159833; doi:10.7150/thno.105894)
Supplement: Supplementary file 1 — Supplementary figures and tables. [file thnov15p6257s1.pdf]

# Supplementary materials

**Supplement Table 1: The sequences for genotyping primers were as follows (5'- 3')**

| Genes (Mouse): | Forward (5'-3'): | Reverse (5'-3'): |
|---|---|---|
| BCKDK | CCTCTCCATCTTCTTAATGCTGGG | GTCAGTAATAGGGGGATGGAGAGAT |
| Ubiquitin C Cre | GACGTCACCCGTTCTGTTG | AGGCAAATTTTGGTGTACGG |

**Supplement Table 2: Antibody information**

| Antibodies | Company | Catalogue No. |
|---|---|---|
| GAPDH | Cell Signal Technology | 2118s |
| BCKDHA | Abcam | ab126173 |
| p-BCKDHA (Ser 293) | Abcam | ab200577 |
| BCKDK | Abcam | ab128935 |
| PP2Cm | Abcam | ab135286 |
| AKT | Cell Signal Technology | 9272S |
| p-AKT (Ser 473) | Cell Signal Technology | 9271S |
| p-P70S6K (Thr 389) | Cell Signal Technology | 9234S |
| P70S6K | Cell Signal Technology | 9202S |
| AMPKα | Cell Signal Technology | 2523S |
| p-AMPKα | Cell Signal Technology | 4188S |
| Anti-IgG(H+L) Rabbit | Bio-RAD | 1706515 |
| Anti-IgG(H+L) Mouse | Bio-RAD | 1706516 |

**Supplement Table 3: The sequences for RT-PCR primers were as follows (5'- 3')**

| Genes (Mouse): | Forward (5'-3'): | Reverse (5'-3'): |
|---|---|---|
| 18s | AGGCCCTGTAATTGGAATGAGTC | GCTCCCAAGATCCAACTACGAG |
| BNP | GAAGGTGCTGTCCCAGATGATT | GCTCTGGAGACTGGCTAGGACTT |
| ANP | AGGCAGTCGATTCTGCTTGA | CGTGATAGATGAAGGCAGGAAG |
| CRP | AGCTACTCTGGTGCCTTCTG | GAGAAGACACTGAAGCTGCG |
| Col1a1 | CGATGGATTCCCGTTCGAGT | GAGGCCTCGGTGGACATTAG |
| Col3a1 | GAGAGCGAGGCCTTCCCGGA | GGGAGCCAGCGGGACCTTGT |
| BCAT2 | CTCATCCTGCGCTTCCAG | CACACCCGAAACATCCAATC |
| BCKDHA | CAGTCCCGCAGGAAGGTGA | TAGTGCTCCCCGTAGGTCTGC |
| BCKDHB | GCAGTGGAACAGGTCCCAGTAG | TATCCACATCCCAAGGCACAAT |
| DBT | GCTCAGGAAAAGATGGCAGAA | TTTGGGCTGTGGTGGAGGT |
| PPM1K | TCTCATTGGCAAACGGAAAG | CAGACAGGTGGGCATAACTCG |
| BCKDK | GCTTCCGTAGCCTTCCTTT | GGTGAGTAGCCAGCATTCG |

## Figure Legend S1-S9

**Figure. S1 HFpEF mice exhibit diastolic dysfunction. A-C,** Echocardiography analysis of fraction shortening (FS) (A) and Isovolumic relaxation time (IVRT) (B) and Mitra Valve E/A ratio (C) in Control and HFpEF two-hit treated mice at indicated time points. n = 6-17. Two-way ANOVA followed by Turkey's test was used in A-C.

**Figure. S2 HFpEF mice exhibit cardiac fibrotic remodeling.** H&E staining for cardiac section and Masson's trichrome staining for left ventricle section in Control and HFpEF groups. Scale bars, 600µm (H&E) and 50µm (Masson).

**Figure. S3 HFpEF mice exhibit cardiac hypertrophic remodeling and nitrosative stress. A-C,** Real-Time PCR analysis of relative mRNA expression of BNP (A), CRP (B) and Col1a1 (C) in control and HFpEF mice left ventricle samples. n = 5-9. **D-E,** S-nitrosylation blot (D) and quantification (E) for left ventricle tissues in Control and HFpEF groups. n = 5. Student t-test was used for statistical analysis.

**Figure. S4 HFpEF mice exhibit changes in BCAA catabolism. A-F,** Circulating BCAA and BCKA levels at 8 (A-B), 11 (C-D) and 13 (E-F) weeks post HFpEF stimulation. n = 5-8. **G,** Relative mRNA expression of *Bcat2*, *Bckdha*, *Bckdhb*, *Dbt*, *Ppm1k* and *Bckdk* in Control and HFpEF mice left ventricle samples. n = 5-8. **H-I,** Relative protein level of BCKDK, PP2Cm and GAPDH (H), and quantification of p-BCKDHA (Ser 293), BCKDHA, BCKDK and PP2Cm (I) in control and HFpEF mice left ventricle samples. **J,** Summary schematic of differences in BCAA metabolites and BCAA catabolic enzymes in HFpEF compared to Control. One-way ANOVA followed by Turkey's test was used in A-F, G and I.

**Figure. S5 Characterization of BCKDK$^{flox/flox}$ and BCKDK-uKO mice at baseline. A**, Schematic representation of global BCKDK knockout mice generated using ubiquitin-Cre (u-Cre). **B**, Real-Time PCR analysis of BCKDK mRNA expression in metabolic organs of BCKDK$^{flox/flox}$ and BCKDK-uKO mice at baseline. **C-E**, Western blot analysis of p-BCKDHA (Ser 293), BCKDHA and their corresponding GAPDH as loading control in liver (C), kidney (D) and skeletal muscle (E) of BCKDK$^{flox/flox}$ and BCKDK-uKO mice at baseline. **F-I**, Fat mass (F), Lean mass (G), Body Weight (H) and Glucose tolerance test (GTT) (I) of BCKDK$^{flox/flox}$ and BCKDK-uKO mice at baseline. **J-N**, Echocardiography analysis of left ventricle ejection fraction (J), Fraction Shortening (K), IVRT (L), E/e' (M) and E/A ratio (N) in BCKDK$^{flox/flox}$ and BCKDK-uKO mice at 20-24 weeks of age at baseline. **O-R**, Real-Time PCR analysis of mRNA expression of ANP (O), BNP (P), Col1a1 (Q) and Col3a1 (R) in BCKDK$^{flox/flox}$ and BCKDK-uKO mice at 20-24 weeks of age at baseline. **S-T**, Masson's trichrome staining (S) for left ventricle section and H&E staining (T) for cardiac section in BCKDK$^{flox/flox}$ and BCKDK-uKO mice at 20-24 weeks of age at baseline. **U-V**. Serum BCAA

(U) and BCKA (V) levels in control and BCKDK-uKO mice at baseline. Repetitive t-test was used for I. Student t-test was used in F-H, J-R, U-V.

**Figure. S6 Cardiac BCKAs and corresponding derivatives levels in BCKDK$^{flox/flox}$ and BCKDK-uKO mice post HFpEF stimulation**. **A**. Heart BCKA levels in control and BCKDK-uKO HFpEF mice. **B**. Heart 2-hydroxy-3-methylvaleric acid level in control and BCKDK-uKO HFpEF mice. **C**. Heart 2-hydroxyisocaproate level in control and BCKDK-uKO HFpEF mice. Student t-test was used in B-C.

**Figure. S7 Targeting BCKDK to accelerate BCAA catabolism has no impact on blood pressure. A,** Schematic view of experimental design. BCKDK$^{flox/flox}$ and BCKDK-uKO mice were fed with tamoxifen and treated with BT2 or vehicle, blood pressure was measured by telemetry, Average daily pressure over the course of telemetry experiment from light cycle (7:00-19:00) and dark cycle (19:00-7:00). **B-C,** Systolic blood pressure measured via telemetry in BCKDK$^{flox/flox}$ and BCKDK-uKO mice before (B) and after (C) tamoxifen treatment. n = 4-8. **D,** Systolic blood pressure measured via telemetry in BCKDK$^{flox/flox}$ and BCKDK-uKO mice before and after BT2 treatment. n = 4-8. **E-F,** Diastolic blood pressure measured via telemetry in BCKDK$^{flox/flox}$ and BCKDK-uKO mice before (E) and after (F) tamoxifen treatment. n = 4-8. **G,** Diastolic blood pressure measured via telemetry in BCKDK$^{flox/flox}$ and BCKDK-uKO mice before and after BT2 treatment. n = 4-8. BT2 was administered via oral gavage once a day at 18:00. Two-way ANOVA followed by Turkey's test was used for B-G.

**Figure. S8 Serum and cardiac KIV downstream metabolites in HFpEF mice with or without BT2 treatment. A-B**. Serum 3-hydroxyisobutyrate (3-HIB) (A) and 3-aminoisobutyric acid (B) levels in BT2 or vehicle treated mice post HFpEF stimulation. **C-D**. Cardiac 3-hydroxyisobutyrate (3-HIB) (C) and 3-aminoisobutyric acid (D) levels in BT2 or vehicle treated mice post HFpEF stimulation. Student t-test was used in A-D.

**Figure. S9 Pharmacological activation of BCAA catabolism prevents cardiac dysfunction and nitrosylative injury. A,** Echocardiography analysis of Fraction Shortening in HFpEF mice treated with BT2 or vehicle. **B-C,** Western blot analysis (B) and quantification (C) of s-nitrosylation level in HFpEF mice treated with BT2 or vehicle. Two-way ANOVA followed by Turkey's test was used for A. Student t-test was used in C.

# Figure S1

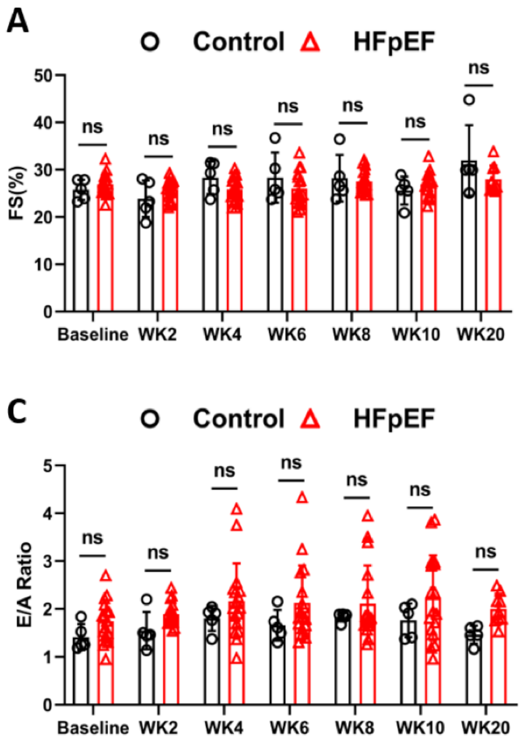
A
Control
HFpEF
FS(%)
Baseline
WK2
WK4
WK6
WK8
WK10
WK20
ns
B
IVRT(ms)
P<0.001
P=0.009
P<0.0001
P=0.0006
C
E/A Ratio

Figure S2

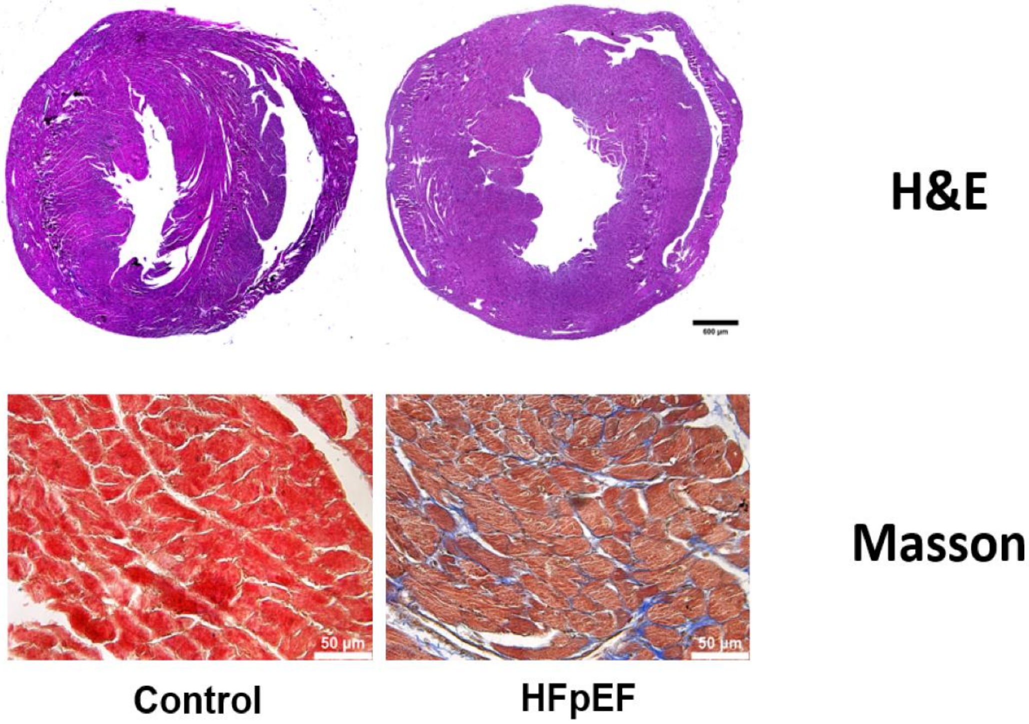

**Figure S3**

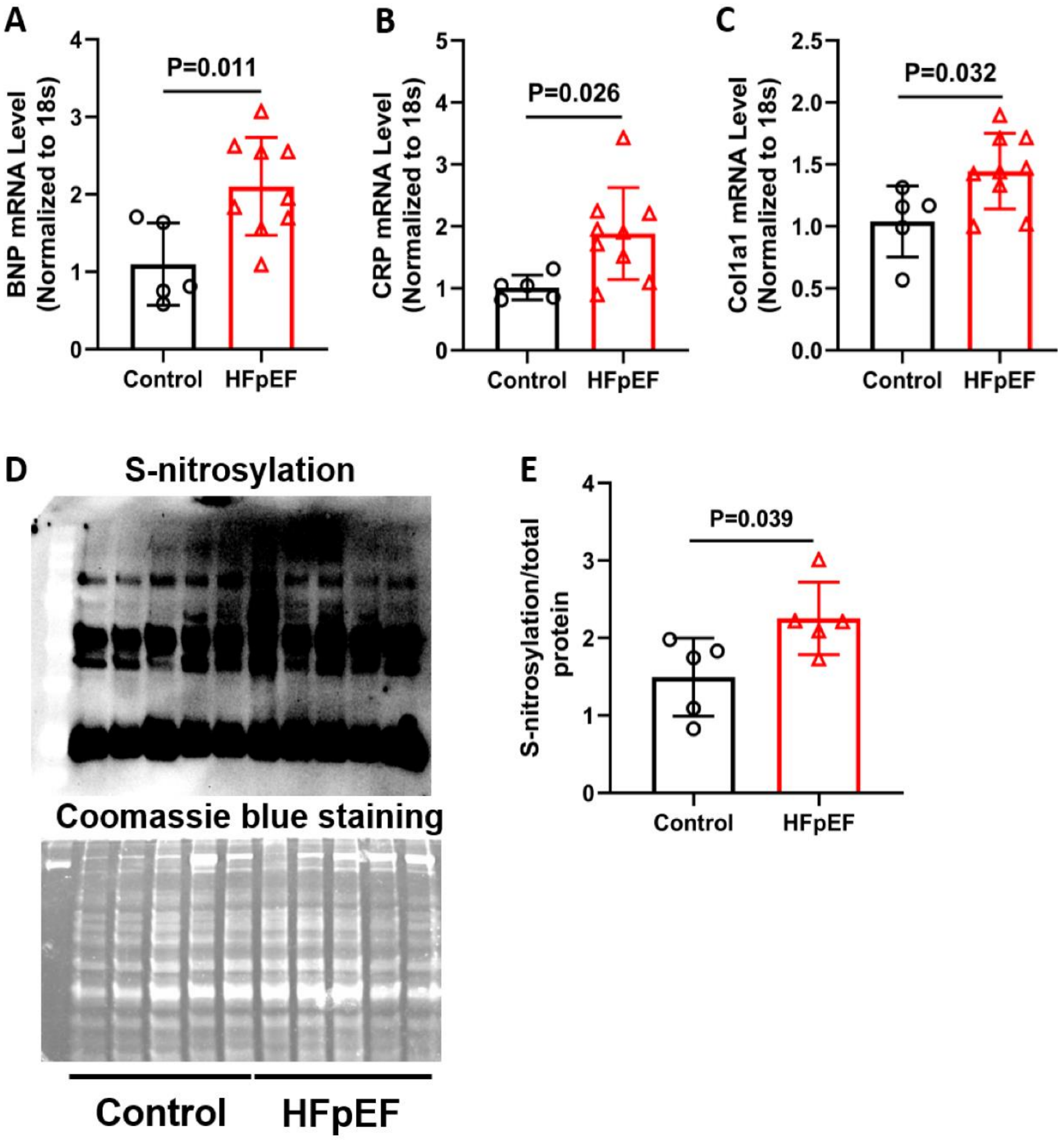

A
BNP mRNA Level (Normalized to 18s)
P=0.011
0
1
2
3
4
Control
HFpEF
B
CRP mRNA Level (Normalized to 18s)
P=0.026
0
1
2
3
4
5
Control
HFpEF
C
Col1a1 mRNA Level (Normalized to 18s)
P=0.032
0.0
0.5
1.0
1.5
2.0
2.5
Control
HFpEF
D
S-nitrosylation
Coomassie blue staining
Control
HFpEF
E
S-nitrosylation/total protein
P=0.039
0
1
2
3
4
Control
HFpEF

# Figure S4

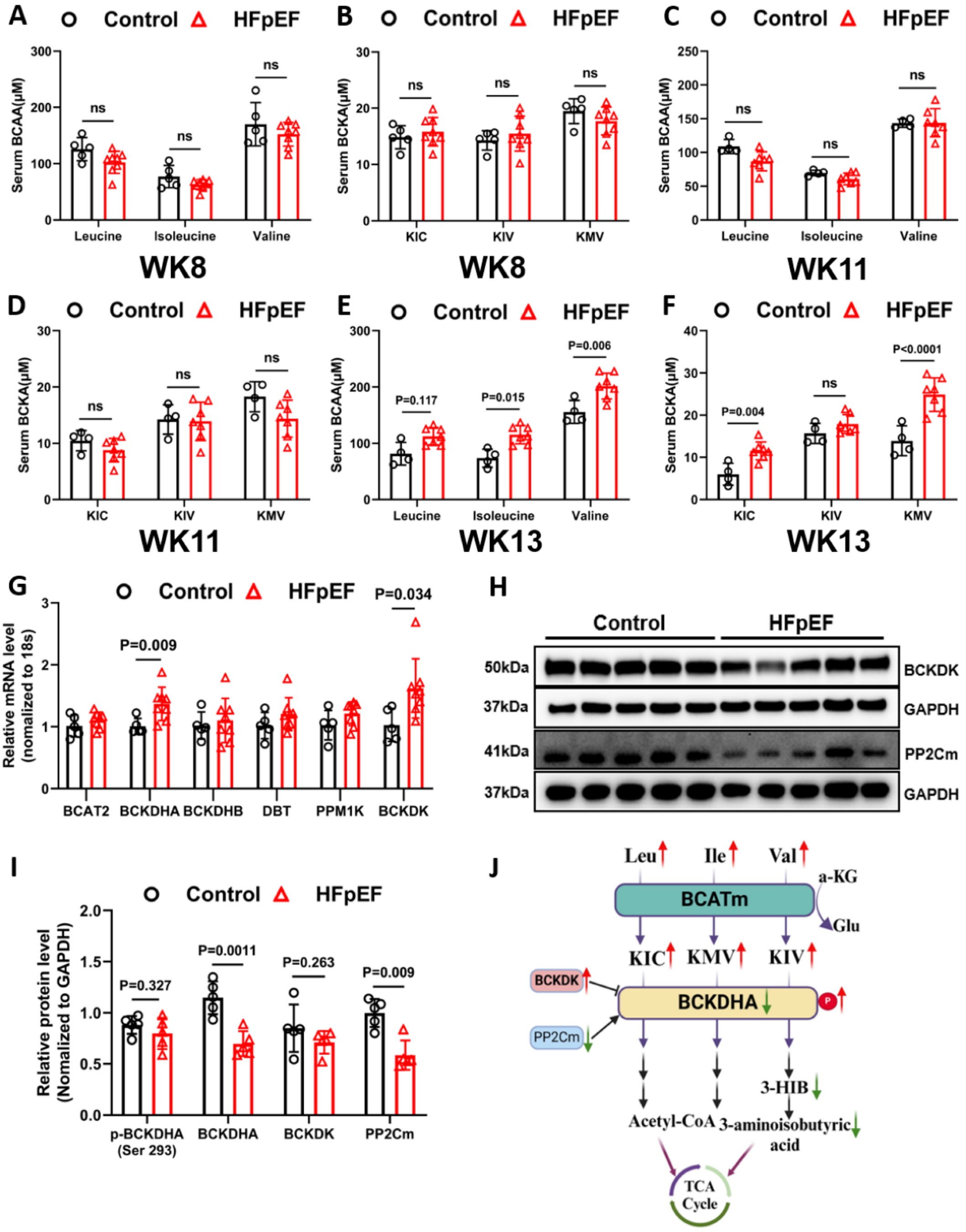

A
Control
HFpEF
Serum BCAA(μM)
Leucine
Isoleucine
Valine
ns
WK8
B
Serum BCKA(μM)
KIC
KIV
KMV
WK8
C
WK11
D
WK11
E
P=0.117
P=0.015
P=0.006
WK13
F
P=0.004
P<0.0001
WK13
G
Relative mRNA level (nomalized to 18s)
P=0.009
P=0.034
BCAT2
BCKDHA
BCKDHB
DBT
PPM1K
BCKDK
H
50kDa
37kDa
41kDa
BCKDK
GAPDH
PP2Cm
I
Relative protein level (Nomalized to GAPDH)
P=0.327
P=0.0011
P=0.263
P=0.009
p-BCKDHA (Ser 293)
J
Leu
Ile
Val
a-KG
BCATm
Glu
KIC
KMV
KIV
BCKDHA
3-HIB
Acetyl-CoA
3-aminoisobutyric acid
TCA Cycle

# Figure S5

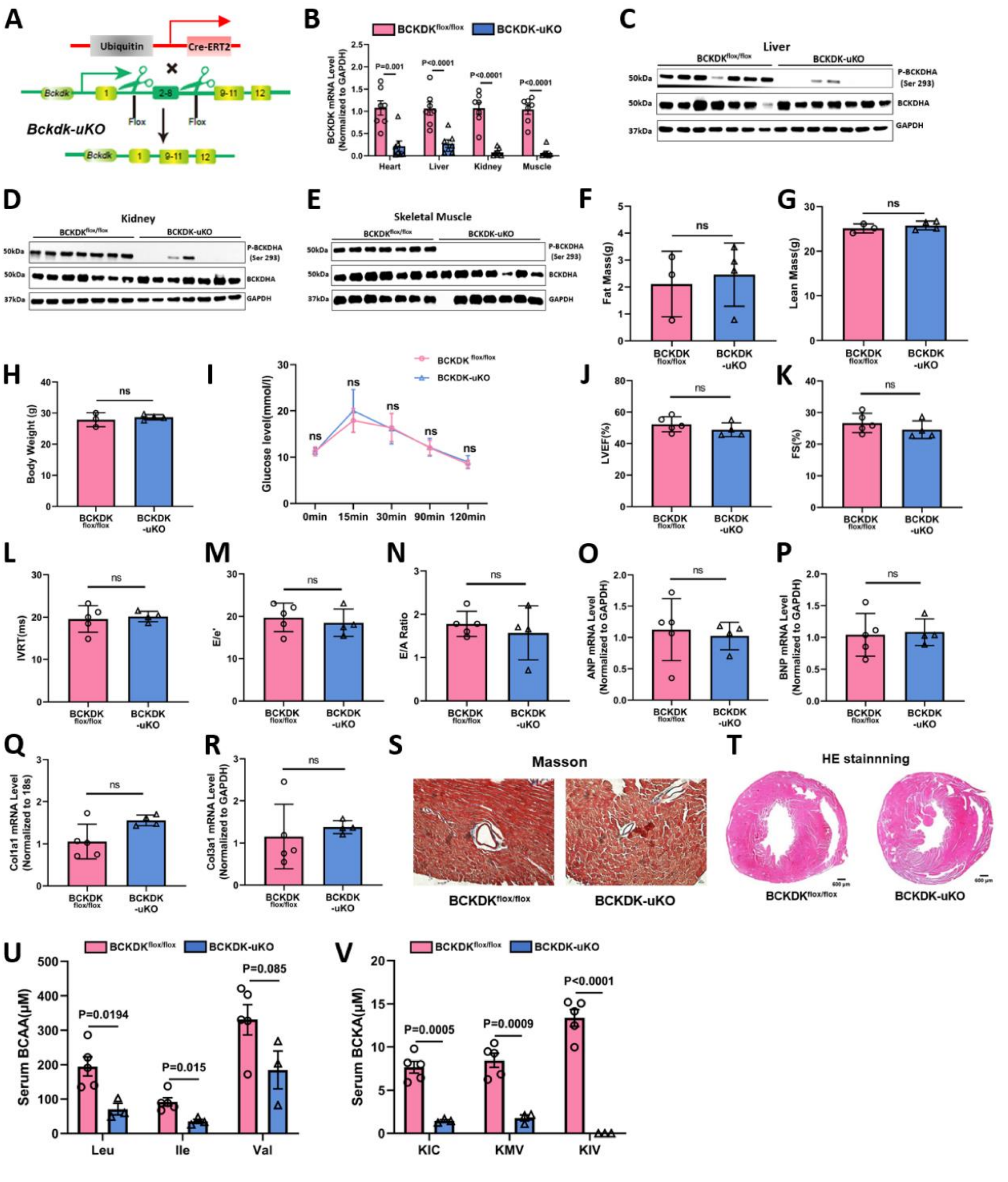

A
Ubiquitin
Cre-ERT2
Bckdk
Flox
Bckdk-uKO
B
BCKDK flox/flox
BCKDK-uKO
BCKDK mRNA Level (Normalized to GAPDH)
P=0.001
P<0.0001
P<0.0001
P<0.0001
Heart
Liver
Kidney
Muscle
C
Liver
BCKDK flox/flox
BCKDK-uKO
P-BCKDHA (Ser 293)
BCKDHA
GAPDH
D
Kidney
E
Skeletal Muscle
F
Fat Mass(g)
G
Lean Mass(g)
H
Body Weight (g)
I
Glucose level(mmol/l)
0min
15min
30min
90min
120min
J
LVEF(%)
K
FS(%)
L
IVRT(ms)
M
E/e'
N
E/A Ratio
O
ANP mRNA Level (Normalized to GAPDH)
P
BNP mRNA Level (Normalized to GAPDH)
Q
Col1a1 mRNA Level (Normalized to 18s)
R
Col3a1 mRNA Level (Normalized to GAPDH)
S
Masson
T
HE stainnning
U
Serum BCAA(μM)
P=0.0194
P=0.015
P=0.085
Leu
Ile
Val
V
Serum BCKA(μM)
P=0.0005
P=0.0009
P<0.0001
KIC
KMV
KIV

# Figure S6

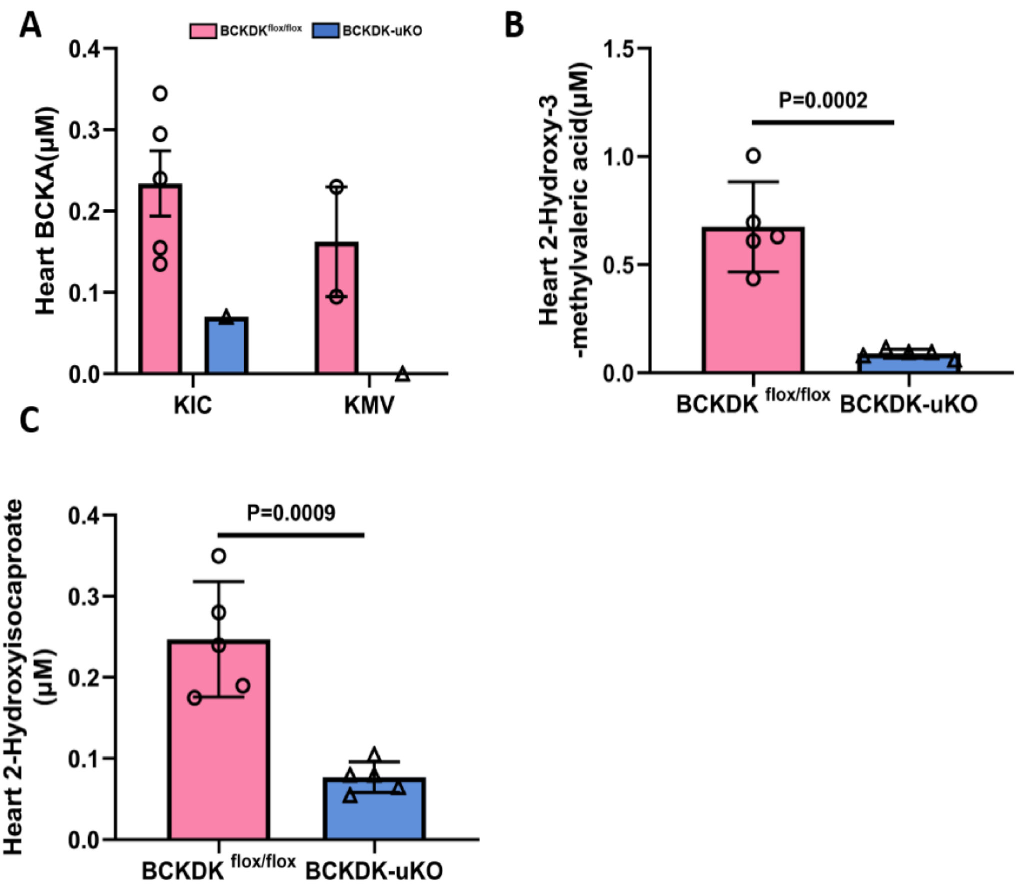

A
BCKDKflox/flox
BCKDK-uKO
Heart BCKA(μM)
0.4
0.3
0.2
0.1
0.0
KIC
KMV
B
Heart 2-Hydroxy-3 -methylvaleric acid(μM)
1.5
1.0
0.5
0.0
P=0.0002
BCKDK flox/flox
BCKDK-uKO
C
Heart 2-Hydroxyisocaproate (μM)
0.4
0.3
0.2
0.1
0.0
P=0.0009
BCKDK flox/flox
BCKDK-uKO

**Figure S7**

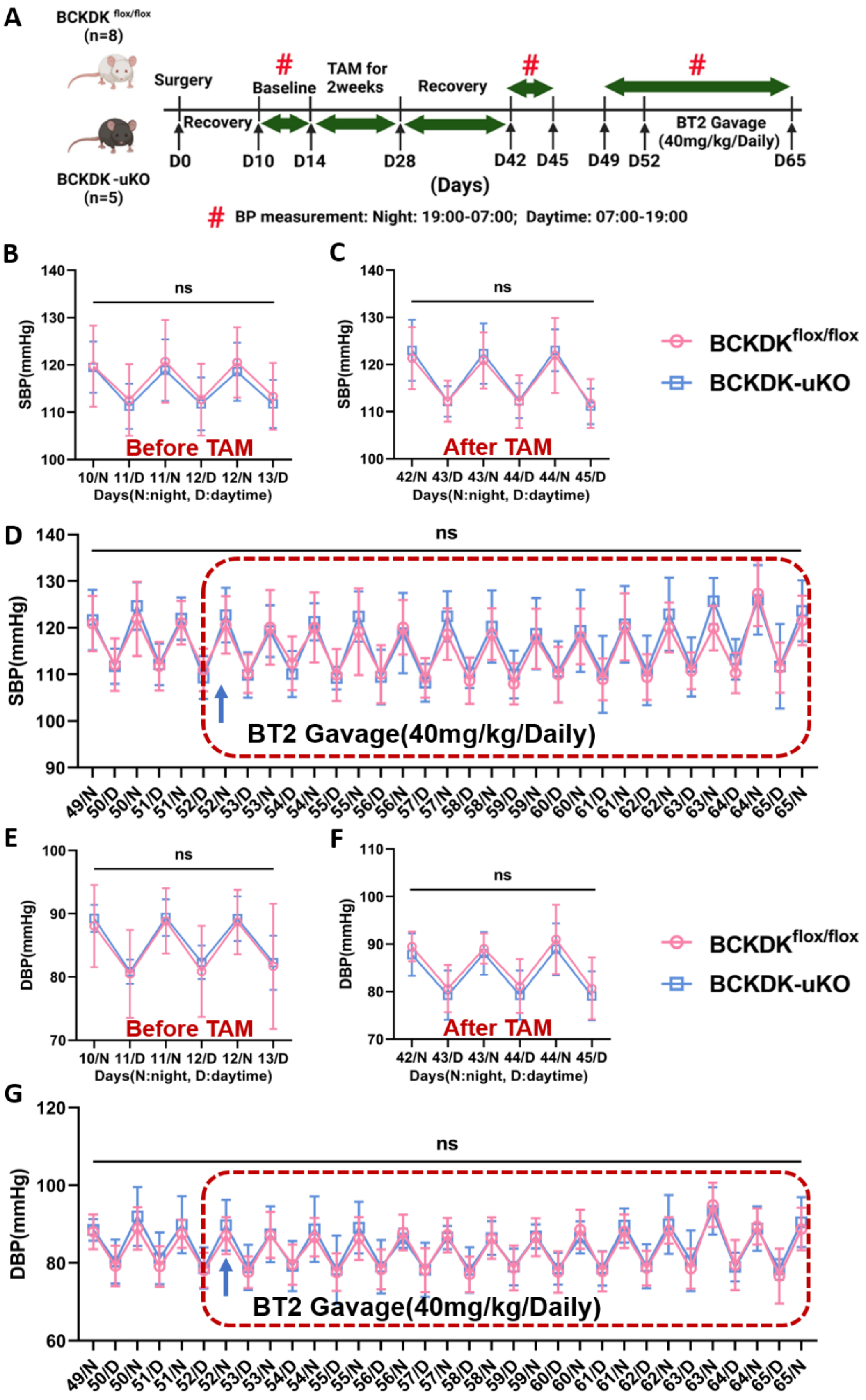

A
BCKDK flox/flox (n=8)
BCKDK-uKO (n=5)
Surgery
Recovery
Baseline
TAM for 2weeks
Recovery
BT2 Gavage (40mg/kg/Daily)
D0
D10
D14
D28
D42
D45
D49
D52
D65
(Days)
# BP measurement: Night: 19:00-07:00; Daytime: 07:00-19:00
B
SBP(mmHg)
ns
Before TAM
10/N 11/D 11/N 12/D 12/N 13/D
Days(N:night, D:daytime)
C
SBP(mmHg)
ns
After TAM
42/N 43/D 43/N 44/D 44/N 45/D
Days(N:night, D:daytime)
BCKDK flox/flox
BCKDK-uKO
D
SBP(mmHg)
ns
BT2 Gavage(40mg/kg/Daily)
49/N 50/D 50/N 51/D 51/N 52/D 52/N 53/D 53/N 54/D 54/N 55/D 55/N 56/D 56/N 57/D 57/N 58/D 58/N 59/D 59/N 60/D 60/N 61/D 61/N 62/D 62/N 63/D 63/N 64/D 64/N 65/D 65/N
E
DBP(mmHg)
ns
Before TAM
10/N 11/D 11/N 12/D 12/N 13/D
Days(N:night, D:daytime)
F
DBP(mmHg)
ns
After TAM
42/N 43/D 43/N 44/D 44/N 45/D
Days(N:night, D:daytime)
BCKDK flox/flox
BCKDK-uKO
G
DBP(mmHg)
ns
BT2 Gavage(40mg/kg/Daily)
49/N 50/D 50/N 51/D 51/N 52/D 52/N 53/D 53/N 54/D 54/N 55/D 55/N 56/D 56/N 57/D 57/N 58/D 58/N 59/D 59/N 60/D 60/N 61/D 61/N 62/D 62/N 63/D 63/N 64/D 64/N 65/D 65/N

**Figure S8**

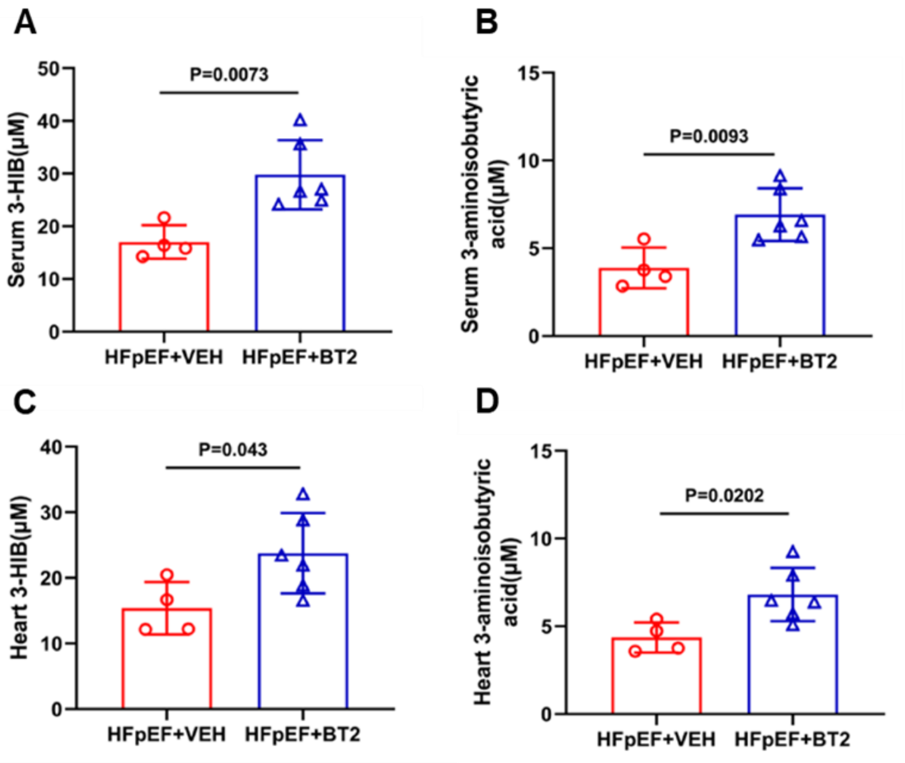
A
Serum 3-HIB(μM)
P=0.0073
HFpEF+VEH
HFpEF+BT2
B
Serum 3-aminoisobutyric acid(μM)
P=0.0093
HFpEF+VEH
HFpEF+BT2
C
Heart 3-HIB(μM)
P=0.043
HFpEF+VEH
HFpEF+BT2
D
Heart 3-aminoisobutyric acid(μM)
P=0.0202
HFpEF+VEH
HFpEF+BT2

**Figure S9**

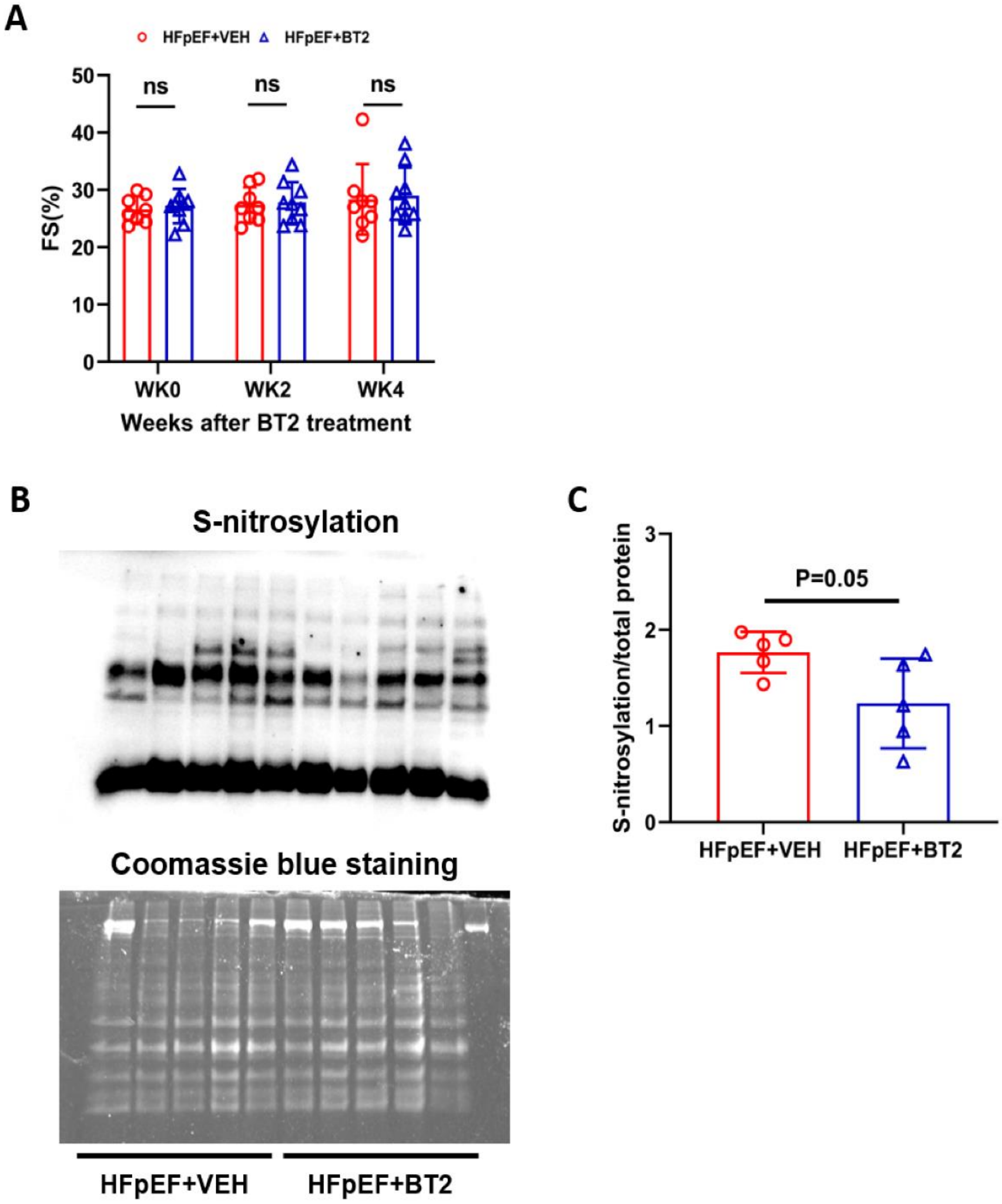
A
HFpEF+VEH
HFpEF+BT2
ns
ns
ns
50
40
30
20
10
0
FS(%)
WK0
WK2
WK4
Weeks after BT2 treatment
B
S-nitrosylation
Coomassie blue staining
HFpEF+VEH
HFpEF+BT2
C
S-nitrosylation/total protein
3
2
1
0
P=0.05
HFpEF+VEH
HFpEF+BT2
